# A Whole-Body Model for Glycogen Regulation Reveals a Critical Role for Substrate Cycling in Maintaining Blood Glucose Homeostasis

**DOI:** 10.1371/journal.pcbi.1002272

**Published:** 2011-12-01

**Authors:** Ke Xu, Kevin T. Morgan, Abby Todd Gehris, Timothy C. Elston, Shawn M. Gomez

**Affiliations:** 1 Department of Biomedical Engineering, University of North Carolina School of Medicine, Chapel Hill, North Carolina, United States of America; 2 Department of Pharmacology, University of North Carolina School of Medicine, Chapel Hill, North Carolina, United States of America; 3 Old Dogs in Training, Carrboro, North Carolina, United States of America; 4 Department of Mathematics, Broome Community College, Binghamton, New York, United States of America; 5 Department of Computer Science, University of North Carolina at Chapel Hill, Chapel Hill, North Carolina, United States of America; Institute for Systems Biology, United States of America

## Abstract

Timely, and sometimes rapid, metabolic adaptation to changes in food supply is critical for survival as an organism moves from the fasted to the fed state, and vice versa. These transitions necessitate major metabolic changes to maintain energy homeostasis as the source of blood glucose moves away from ingested carbohydrates, through hepatic glycogen stores, towards gluconeogenesis. The integration of hepatic glycogen regulation with extra-hepatic energetics is a key aspect of these adaptive mechanisms. Here we use computational modeling to explore hepatic glycogen regulation under fed and fasting conditions in the context of a whole-body model. The model was validated against previous experimental results concerning glycogen phosphorylase a (active) and glycogen synthase a dynamics. The model qualitatively reproduced physiological changes that occur during transition from the fed to the fasted state. Analysis of the model reveals a critical role for the inhibition of glycogen synthase phosphatase by glycogen phosphorylase a. This negative regulation leads to high levels of glycogen synthase activity during fasting conditions, which in turn increases substrate (futile) cycling, priming the system for a rapid response once an external source of glucose is restored. This work demonstrates that a mechanistic understanding of the design principles used by metabolic control circuits to maintain homeostasis can benefit from the incorporation of mathematical descriptions of these networks into “whole-body” contextual models that mimic *in vivo* conditions.

## Author Summary

Homeostasis of blood glucose concentrations during circadian shifts in survival-related activities, sleep and food availability is crucial for the survival of mammals. This process depends upon glucose intake, short-term storage as glycogen, and gluconeogenesis. The integration of hepatic glycogen anabolic and catabolic dynamics with whole body energetics is critical for survival. In this paper we use computational modeling to investigate the potential survival advantage of substrate (futile) cycling of glycogen and glycogen precursors. Our simulations, combined with published experimental results of other researchers, indicate that as the body enters a state of fasting, the activity of enzymes involved in the synthesis of glycogen increases leading to increased substrate cycling. This increase in substrate cycling allows the system to respond more rapidly once new external sources of glucose become available. The whole-body computational model developed for this work allows the metabolic control circuitry to be studied under simulated in vivo conditions, providing functional insights that are not evident when individual modules of glycogen regulatory circuitry are examined in isolation.

## Introduction

Glucose is the major metabolic fuel of mammals, with its maintenance at appropriate levels within the body being crucial for normal function, while dysregulation is associated with diseases such as diabetes mellitus, galactosemia and glycogen storage diseases [1]. Maintaining glucose levels requires a highly responsive control system capable of balancing a wide range of environmental conditions, perhaps the most basic of which is managing the uptake of nutrients from food at irregular time intervals. Specifically, transitions between fed and fasted states require rapid shifting between the storage of excess glucose, in the form of glycogen, within the liver and muscle and the breakdown of these stores for delivery of glucose to other organs. In healthy individuals, proper functioning of this system ensures that available nutrients are efficiently captured and stored during times of excess, while effectively managed and distributed during times of fasting.

The rate with which the organism responds to these changes can play a critical role in survival. Optimization of energy storage is essential during competition for sparse food supplies, while rapid delivery of these energy supplies during hasty retreat from predators can mean the difference between life and death [2]. A key player in energetics, especially for erythrocyte and brain function, is blood glucose concentration.

The liver is the central organ for regulation of glucose and glycogen and acts as the primary distributor of nutrients through the blood to other tissues. When in a fasted state, the liver breaks down glycogen stores, producing glucose for other tissues. After a meal, the liver switches to a glucose consuming state, capturing nearly 26% of the glucose presented to it by the portal system during the first passage [3]. Nearly 10–15% [4], [5] of liver weight is comprised of glycogen stores when filled.

Glucose regulation within the liver is performed by the glycogen circuit that controls both the storage of glucose as glycogen (glycogenesis) as well as its breakdown into glucose-6-phosphate from hepatic stores (glycogenolysis). Of significance is the fact that glycogenolysis and glycogenesis are not the result of a single reversible reaction, but rather are two separate, highly-regulated pathways. Two key molecular players within these pathways are glycogen synthase (GS) and glycogen phosphorylase (GP). GS drives the synthesis of glycogen, with its activity regulated through multiple mechanisms including allosteric activation, covalent modification, as well as enzymatic translocation [6]–[8]. GP catalyzes the rate-limiting step in glycogenolysis and it too, is actively regulated through phosphorylation at a single residue on the NH2 terminus as well as through allosteric regulation [6]–[8]. Both these enzymes exist in activated (GSa and GPa) as well as inactivated (GSb and GPb) states.

**Figure 1:**
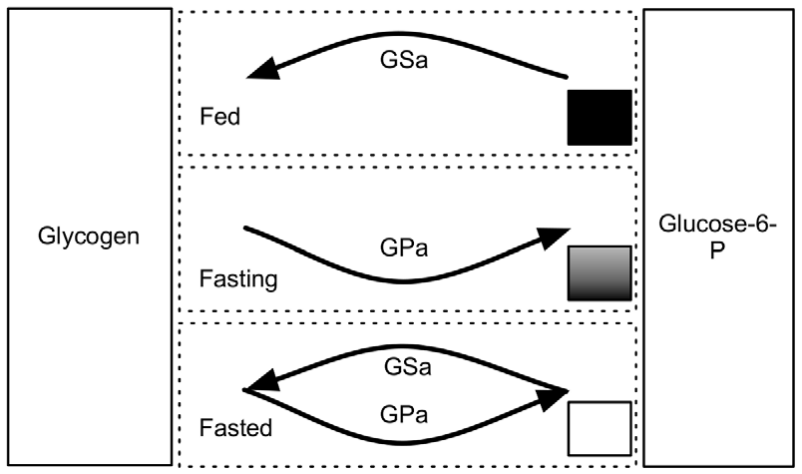
Liver glycogen levels control circuit architecture. Glycogen is synthesized by GSa and broken down into glucose-6-phosphate by GPa. Glycogen levels within the liver are shown in the Fed, Fasting and Fasted state as shaded boxes, with full liver glycogen stores being shown as a solid black box in the Fed portion of the circuit. Arrows indicate which branch of the pathway is active. Substrate cycling occurs in the glycogen-depleted (empty box), Fasted state.

As the synthesis of glycogen and its breakdown into glucose occur through separate pathways, there is the potential for substrate cycling to occur, wherein glucose and glycogen are continuously interconverted. In fact, the glycogen circuit exhibits different behaviors depending on the state of liver glycogen levels (Figure 1). In the fed state, glucose is plentiful in the blood and glycogen levels within the liver are relatively high, resulting in the activation of GS and the synthesis of glycogen. When a fasting state is entered, glycogen levels in the liver are high and blood glucose levels are maintained by the breakdown of this glycogen into glucose-6-phosphate by GPa. Finally, when in the fully fasted state, glycogen stores within the liver are essentially depleted. It is here, in the context of glycogen depletion, that cycling is observed between glycogen and glycose-6-phosphate [9], [10].

It has long been suggested that substrate cycling is a generic mechanism that can potentially improve such properties as sensitivity and system response time, allowing net synthesis when there is a small offset in the substrate concentrations [7], [10]–[13]. However, demonstrations of cycling and its functional relevance in a physiological context are still relatively rare. In this work, we were particularly interested in investigating the potential role of the cycling - no cycling architecture of the glycogen circuit manifested during the transition from a fed to a fasted state. While the benefit of preventing substrate cycling is apparent since energy is dissipated in the form of heat during this process, it is not clear why it is beneficial for glycogen to cycle under the fasted state, as shown in Figure 1.

Mathematical models, which provide one way to explore such questions, have been applied successfully to many biological fields, but their application has been limited in the case of the nutritional sciences [14]. The number of mathematical models of hepatic energy metabolism, as it relates to hepatic glycogen storage, has been slowly increasing in response to interest in the impact of exercise on energetics in the case of diabetes [14], diet [15] and athletic training [16]. In addition, large-scale reconstructions of metabolism, typically based on flux or constraint-based models, have recently been developed for multiple organ systems including the liver [17]–[21]. These stochiometry-based approaches can be used to analyze the relevant biological network solely based upon systemic mass-balance and reaction capacity constraints when kinetic information is missing [22], [23]. However, as these approaches are based on steady state assumptions and do not consider specific kinetic properties, they provide a fundamentally different view of metabolism and metabolic dynamics than detailed mechanistic models.

In the absence of a suitable model for the present work, we developed a physiological model based on a central control glycogen circuitry by Hers et al. [7] and Mutalik et al. [24], with the whole body bioenergetics described in [25]–[29] as well as the feedback and feed forward control loops described in [30]–[33] for maintaining glucose homeostasis under different fed-fasting conditions. We placed specific emphasis on investigating the role of the cycling - no cycling architecture in metabolic functions. Building on previous biochemical and quantitative modeling descriptions, this model embedded the glycogen circuit of the liver within a physiological system composed of muscle, adipose tissue and blood compartments. By controlling the glucose injection rate into the blood stream, we were able to simulate system response across a broad range of fed/fasting conditions. Our simulation results reproduced previously published experimental observations and further indicated that the cycling design in Figure 1 provides a mechanism for decreasing the amount of time it takes to convert glucose to glycogen in the fasted state.

## Results/Discussion

### Model overview

**Figure 2:**
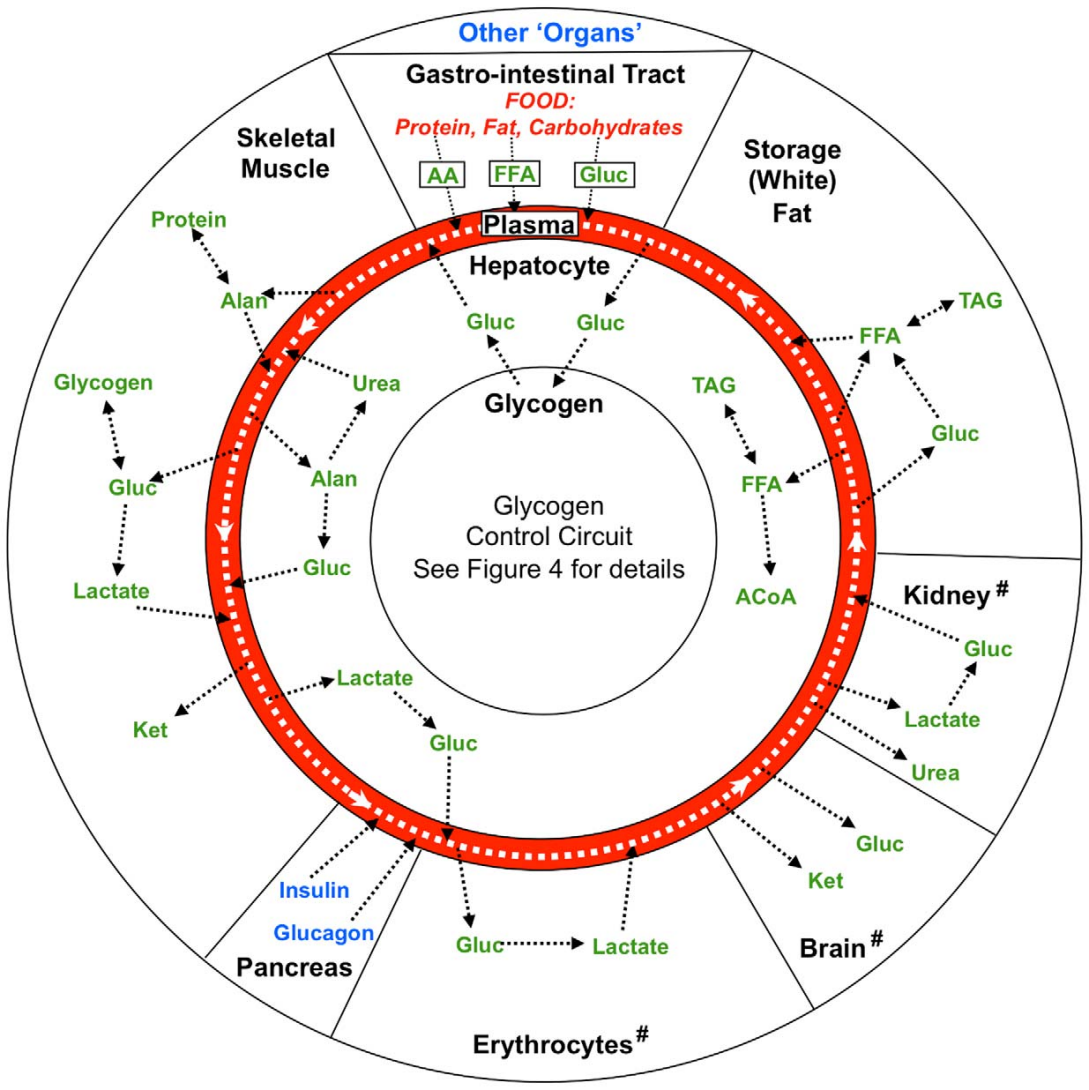
Diagrammatic representation of key features of the physiological model. The general design principles of the model are based on established bioenergetic physiology [1]. The liver, placed at the center of this diagrammatic representation of “the body”, contains the glycogen circuitry which lies within hepatocytes connected to other organs by the vascular system (show in red). Blood within the vascular system travels around the body, carrying materials between the liver and other organs, with a cycle time of about one minute. Key: Gluc = glucose; FFA = free fatty acids; Ket = ketones; TAG = triacylglycerol; ACoA = acetyl CoA; Alan = alanine. Note that kidney, brain and erythrocytes are not included in the current model.

We now give a brief overview of our model, with full details and the complete set of model equations provided in Protocol S1. Note that the complete MATLAB package together with the description file are provided in Protocol S2 and S3, respectively. The SBML code is also provided in Protocol S4 for a broader usage and implementation. As noted earlier, glycogen is created from glucose during feeding and is subsequently degraded to release glucose-6-phosphate during fasting. The hepatic glycogen circuit controlling this process is embedded within the hepatocyte at the center of our physiological model (Figure 2). Blood is depicted as a closed loop, being carried around the body to connect multiple tissue compartments, including the liver, muscle, and fat. Thus blood functions as a transport system within our model, providing the resources needed to manufacture and store hepatic glycogen during the fed state while carrying its major degradation product, glucose, away during the fasted state for use by other tissues. The liver is currently the most detailed compartment in this model, including selected aspects of glycogenolysis, glycogenesis, glycolysis, gluconeogenesis, the TCA cycle, lipogenesis, lipolysis and ketogenesis (See Protocol S1 for model equations).

As an animal moves through the fed, fasting and fasted states, its body switches to different types of metabolic fuels to stabilize blood glucose concentration. This transition is controlled in large part by the blood levels of insulin and glucagon, both of which are generated in a reciprocal manner by the pancreas in response to changing blood glucose levels. Insulin and glucagon are mutually antagonistic with respect to many aspects of intermediary metabolism and their effects on bioenergetics [25], [34]. Insulin is a key regulator for carbohydrate and fat metabolism in the body. It enhances blood glucose uptake to form triglycerides and glycogen and suppresses pathways such as gluconeogensis and glycogenolysis [35]. Glucagon, on the other hand, is secreted from the pancreas when blood glucose concentration is low. It inhibits glycolysis and stimulates hepatic glycogenolysis and gluconeogenesis in liver by increasing the concentration of cAMP [36]. The elevated level of cAMP in turn activates a cascade of enzymes in the glycogen control circuitry that enhance the degradation of glycogen molecules [7]. Insulin and glucagon, working in a reciprocal fashion, in conjunction with other hormonal regulators, such as leptin and epinephrine, maintain glucose homeostasis in biological systems. Our physiological model also incorporates aspects of the Cori cycle, where lactate from muscle and erythrocytes is carried to the liver and converted to glucose for reuse by these tissues.

Blood glucose is provided from absorbed carbohydrates during feeding up until digestion is complete, at which point hepatic glycogen stores take over this role. Depletion of hepatic glycogen occurs over a period of 12–24 hours, though this varies greatly with activity levels [7], [37]. Once hepatic glycogen stores are consumed, blood glucose levels are maintained by gluconeogenesis. This process uses energy derived from storage fat in the form of acetyl CoA and the carbon skeletons of glycogenic amino acids. In the present physiological model, glycogenic amino acids are represented by alanine derived from muscle. The major sites of gluconeogenesis are the kidney and the liver, with only the latter being represented here. As blood glucose levels fall due to hepatic glycogen depletion, blood insulin levels fall while glucagon levels rise, leading to biochemical changes resulting in the use of alternative fuels in the form of free fatty acids and ketones, and gluconeogenesis which requires the use of such energy as mentioned above.

**Figure 3:**
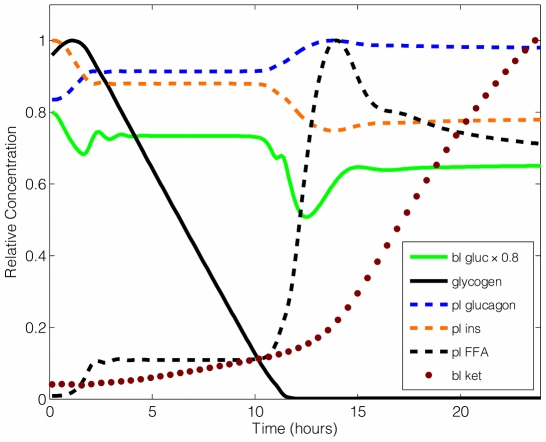
Simulation results: dynamic responses of selected hormones and substrates during a 24-hour fasting period. Note that hormone and substrate concentrations are normalized by their maximum values during the simulation. Blood glucose concentration has a multiplier of 0.8 to give a better view. Key: bl gluc = blood glucose; pl glucagon = plasma glucagon; pl ins = plasma insulin; pl FFA = plasma free fatty acids; bl ket = blood ketone bodies.

In tissues such as the heart and muscle, a number of factors regulate the use of alternative energy sources in order to spare blood glucose for use by erythrocytes (which depends solely on blood glucose [38]) and the brain (which mainly depends on blood glucose but can use ketone bodies as an alternative during fasting [39]). Our general bioenergetic model includes a number of tissue and biochemical components that were selected on the basis of their relationship to glycogen metabolism. The timing of these events and the dynamics of blood insulin, glucagon, glucose, free fatty acids, ketones, and levels of hepatic glycogen stores in response to fasting and feeding cycles from our simulation are shown in Figure 3 and are consistent with those which were previously described in [25]–[29].

### The cyclic-AMP (cAMP) induced glucose-glycogen circuitry

#### Internal cues: cAMP activates a cascade of enzymes

**Figure 4:**
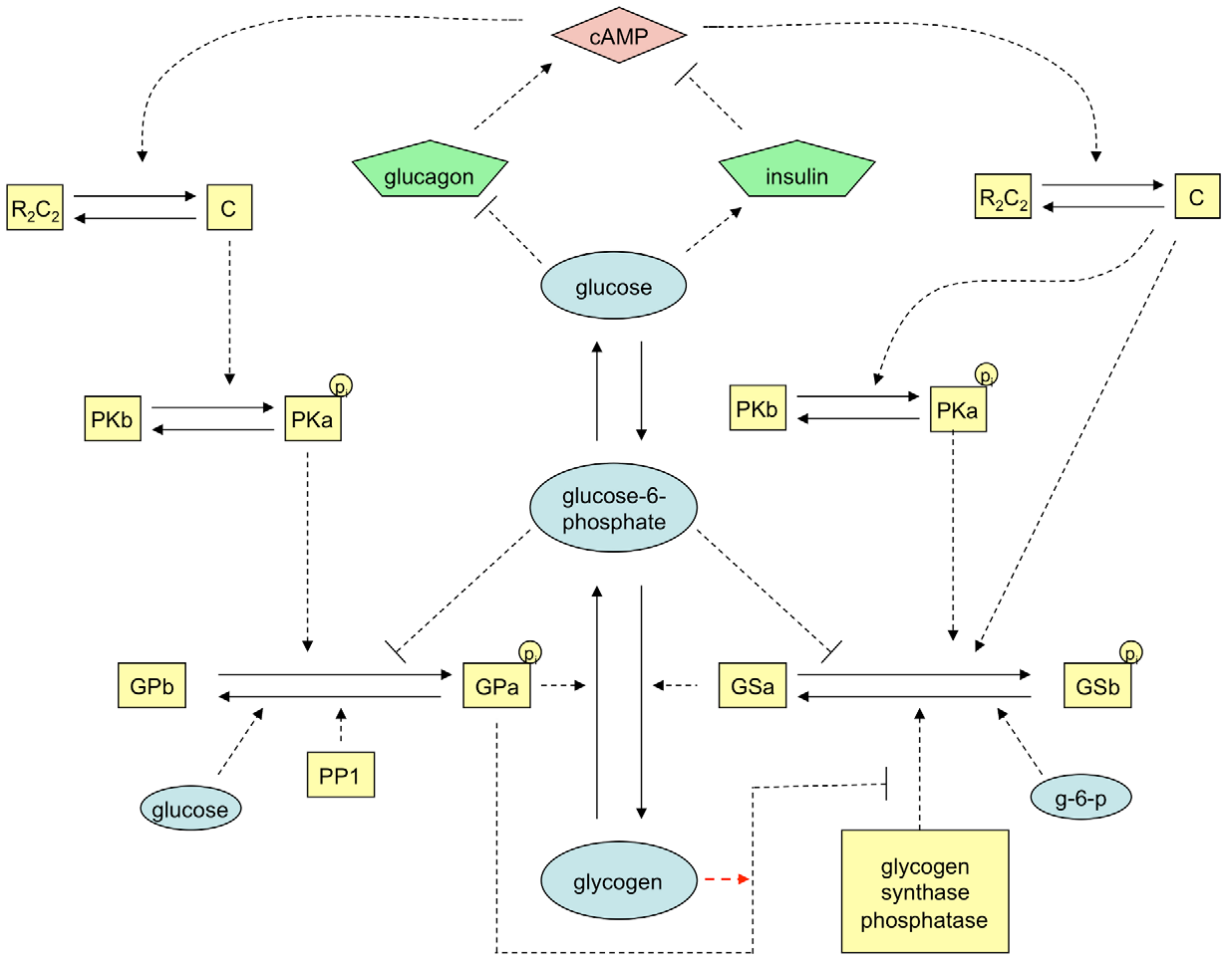
The central control glycogen circuitry (modified from Figure 3.1 in [**43**]). Rectangles and circles enclose the names or abbreviations of enzymes and substrates accordingly. The reactions as a result of an increase in cAMP concentrations are shown with bold arrows. cAMP = cyclic adenosine monophosphate; R2C2 = cAMP dependent protein kinase; C = R2C2 catalytic subunit; PKb = inactive phosphorylase kinase; PKa = active phosphorylase kinase; GPb = inactive glycogen phosphorylase; GPa = active glycogen phosphorylase; GSb = inactive glycogen phosphorylase; GSb = inactive glycogen synthase; GSa = active glycogen synthase; P = phosphate; g-6-p = glucose-6-phosphate; PP1, protein phosphatase-1.

The glycogen circuitry is activated by intra- and extracellular signals including cAMP, glucose and glucose-6-phosphate. As blood glucose begins to fall in the post-absorptive state, glucagon is secreted from the pancreas and causes elevation of cAMP levels [36]. The increase in cAMP signal leads to activation of cAMP-dependent protein kinase (CAPK), which in turn activates phosphorylase kinase (PK). PK phosphorylates GS (less active, b-form) and GP (more active, a-form) and drives the system to enter a catabolic state where glycogen molecules are broken down to supply liver glucose output and maintain blood glucose levels. Note that GP can be inhibited directly by high levels of glucose [10] as indicated in Figure 4. Protein phosphatase-1 (PP-1) is another key element in this regulation, acting as the primary phosphatase catalyzing the dephosphorylation of PK, GP, and GS. A variety of evidence shows that GPa has an inhibitory effect on the dephosphorylation (activation) of GS under fed conditions [7], [40], as shown on the bottom of Figure 4. This inhibition is mediated by the direct binding of GPa to glycogen synthase phosphatase (GS phosphatase), a major enzyme catalyzing the conversion of GS from the D (b - phosphorylated, less active) to the I (a - unphosphorylated, active) form. To model this inhibitory effect, we incorporated the dissociation constant (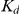) between GPa and GS phosphotase, as first proposed by Mutalik et al. [24]. As a whole, the intracellular cAMP concentration determines the level of activated kinases and phosphatases within the cell, which in turn determines the levels of GPa and GSa and therefore the glycogen degradation and synthesis rates [7], [24].

As mentioned above, GPa inactivates GS phosphatase by direct binding under the fed condition. In fact, Stalmans et al. [40] observed that only after the levels of GPa dropped below 10% of the total enzyme (a+b) would liver GS then be activated. It has also been shown that the inhibition of GS phosphatase by GPa depends on liver glycogen concentration and that a minimal amount of glycogen is required for this inhibition [41], [42]. Therefore, the level of inhibition of GS phosphatase by GPa is highly dependent on both the activation state induced by external glucose and hormonal cues, as well as the internal state of liver glycogen stores (indicated by a red arrow in Figure 4). Since this inhibition is induced by direct binding of GPa to GS phosphatase, one way to model the difference in inhibition is through the dissociation constant (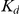) of these two enzymes, as first proposed by Mutalik et al. [24]. Note that in their work, a 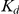 of 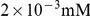 was set to correspond to a fasted state while a 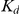 of 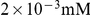 corresponded to a fed state. As described further below, we similarly use the dissociation constant of GS phosphatase and GPa as a means to model the discrepancy in the enzymatic activities in fed and fasting states. For a detailed discussion, we refer the readers to [24].

#### External cues: Insulin, glucagon and cAMP

Insulin and glucagon are two key hormones that both regulate, and are regulated by, blood glucose concentration. The concentrations of these two hormones are governed by the equations [43]:
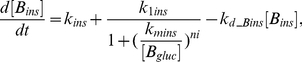
(1)

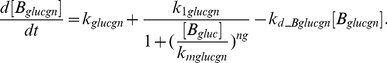
(2)where 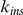 and 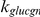 are the basal secretion rates and 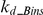 and 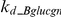 are the degradation rates for insulin and glucagon, respectively. We assume that the release rate of insulin into the blood increases with glucose levels according to Hill kinetics with a Hill coefficient of 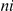. The maximal insulin-induced release rate is 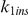 and the glucose concentration at which this rate is half its maximum is 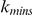. Similarly, to model the decrease in glucagon at high blood glucose levels, we assume the release rate of glucagon decreases according to Hill kinetics. The Hill coefficient is 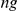, the maximum induced release rate is 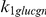, and the glucose concentration at which the release rate is half its maximum is 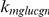. The values of the parameters in the above two equations are chosen such that the concentrations of insulin and glucagon are in the range of 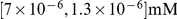 and 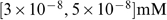, taken from physiologically relevant ranges determined from rodent studies [27].

cAMP is a secondary messenger that is regulated by both insulin and glucagon. Following a drop in blood glucose levels, cAMP activates CAPK according to the following reaction:
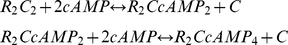
(3)The above reaction includes two steps and results in the release of 2 molecules of catalytic subunit C from CAPK by 4 molecules of cAMP. The equation that governs the concentration of cAMP is then [43]:
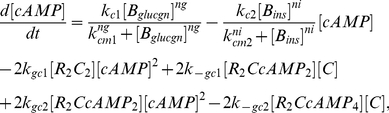
(4)where we assume Michaelis-Menten type kinetics for the regulatory functions of insulin and glucagon on cAMP. The two Michaelis-Menten constants 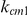 and 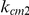 were set to be the mean values of the glucagon and insulin concentrations, 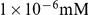 and 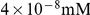, respectively, while the parameters involved in the activation of CAPK are adopted from [24].

The amount of the catalytic subunit C determines the activity level of GP and GS [7], [24]. Together, these two enzymes regulate the metabolism of liver glycogen: if GS is more active, the system converts excessive glucose into glycogen for short-term storage; if GP is mostly active, the system utilizes glycogen to make glucose to supply the needs of other organs. The equation for the glycogen concentration is [43]:
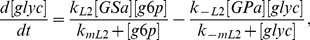
(5)where again we use Michaelis-Menten kinetics to describe the enzymatic activities.

Finally, the glucose concentrations in liver 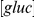 and blood 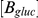 are given in Equations (6) and (7) correspondingly [43], where 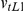, 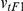 and 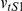 are the glucose transport rates from the blood stream to liver, adipose tissue and muscle, 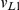 and 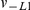 are the reaction rates for the conversion of glucose into g6p and g6p back to glucose, 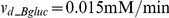 is the degradation rate of blood glucose and 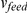 is the feeding function for blood glucose, which is subject to change under different feeding patterns.
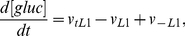
(6)

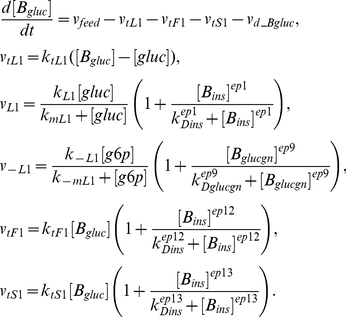
(7)


### The degree of substrate cycling depends upon the dissociation constant 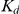


Mutalik and Venkatesh [24] computed the dose response curves of the enzymes in the glycogen circuitry based on their empirically derived input functions for glucose-6-phosphate (G6p) and cAMP. Again, we note that the dissociation constant (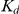) of GS phosphatase and GPa is the key factor in determining the amount of substrate cycling at steady state. In fact, Mutalik et al. [24] defined different physiological states based on the value of 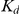, where a smaller value (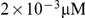) corresponded to a fed state and a larger one (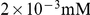) corresponded to a fasted state. We followed a similar approach to construct the dose response curves for these enzymes. Unlike [24], the glycogen circuitry was incorporated into a 4-compartment physiological model. As a result, the dynamics of cAMP and glucose-6-phosphate were simulated directly within our model and the entire system can be more realistically simulated by simply controlling the plasma glucose concentration.

**Figure 5:**
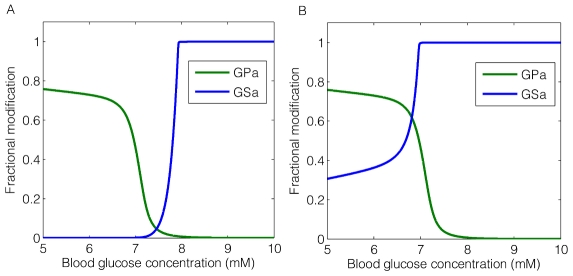
Fractional activation of GPa and GSa plotted against blood glucose concentration under two selected 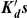
 **.** A: 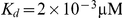 corresponds to a stronger binding between glycogen phosphorylase a (GPa) and GS phosphatase, which results in a strong inhibition on the activation of glycogen synthase (GSa). B: 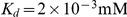 corresponds to a weaker binding between GPa and GS phosphatase, where the inhibition by GPa is partially relieved.

The dose-response curves for GSa and GPa at two specified values of 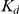 are shown in Figure 5. Here, the system was run to steady state with a fixed blood glucose concentration between 5 mM and 10 mM, the typical range for fed-fasting experiments in rodents [27], [44]–[46]. By increasing the 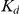 constant from 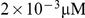 to 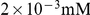, the crossover point of GPa and GSa shifted from a higher glucose concentration to a lower one (from 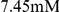 to 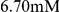) with a correspondingly higher activated fraction (from 5% to 60%). This fraction represents the maximum percentage of both enzymes being active simultaneously, thus it is an indicator of the degree of substrate cycling in the system. We note that the inhibitory effect by GPa on the activation of GS through direct binding to GS phosphatase is partially released with a larger 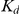.

### The response time for glycogen synthesis decreases with a larger value of 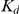 in the glycogen depleted state

From the above discussion, it is apparent that the inhibition of GS activation by GPa through direct regulation of GS phosphatase varies with the state of fasting. With a larger 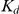, the maximum amount of substrate cycling (co-activated fraction of GS and GP) is higher at the steady state. Here, we further investigated the dynamics of GS and GP but in the context of a glycogen depleted liver.

**Figure 6:**
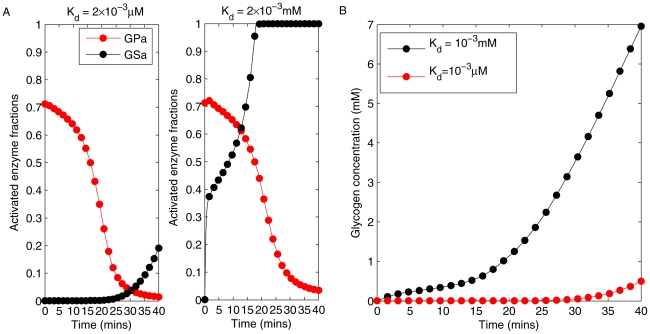
Time evolutions curves of selected enzymes and substrates. A: Time response curves of GSa and GPa under two selected 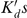 after glucose stimulus enters blood stream at 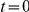 in a glycogen depleted liver. Crossover of GSa and GPa occurs at 13.4 and 30.6 minutes respectively. B: Liver glycogen concentration plotted against time under the two selected 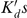.

To simulate the response of the system to glucose in a glycogen depleted state, we provided a constant input of glucose with 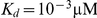 and ran the simulation to steady state. We then gradually throttled back the glucose input, and waited until liver glycogen was completely depleted. Glucose supply then re-entered the blood stream as a step function at t = 0, after which the dynamics of hormone, enzyme and substrate responses were observed. The results for two values of 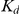, 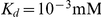 and 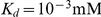 are shown in Figure 6A. Note that the observation period began at 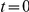. A detailed description of the plasma glucose feeding function 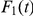 is provided in Figure S5 in Protocol S1.

Figures 6A–B show selected enzyme activities and glycogen concentration as a function of time. Note that “glycogen concentration” here and in the later context refers to the amount of glucose converted to glycogen as one molecule of glycogen comprises an indeterminate number (hundreds or thousands) of glucose subunits. At 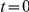, liver glycogen stores are completely depleted and GP is mostly active (over 70% in the *a*-form). The sudden increase in the blood glucose concentration drove the transition from a GP-dominant to GS-dominant scenario. There was a major difference in when and where the intersection of GPa and GSa activity curves occurred for the two selected values of 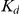. Under 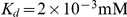, the point of intersection occurred at 60% and 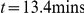 (Figure 6A-left panel). In contrast, this point shifted to 5% and 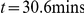 with 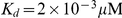 Figure 6B-right panel.

Figure 6B showed the liver glycogen concentration as a function of time. Again, the observations started at 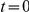 where the blood glucose supply resumed. Readily apparent was the slow but nearly immediate increase in the glycogen concentration at 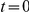 under 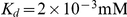 (black line with dots). Recall that at this value, the level of inhibition of GS phosphatase by GPa was much reduced, allowing the coexistence of 60% of GSa and GPa. In contrast, the liver glycogen concentration remained at a negligible level until 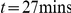 with 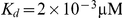 (solid line with squares) where substrate cycling was reduced to 5%. Therefore, the system was able to respond quickly to the glucose stimulus and drive an immediate synthesis of glycogen with a higher level of substrate cycling. In both cases, a dramatic change in the synthesis rate of glycogen occurred where GSa and GPa intersect (15 mins and 30.6 mins correspondingly).

**Figure 7:**
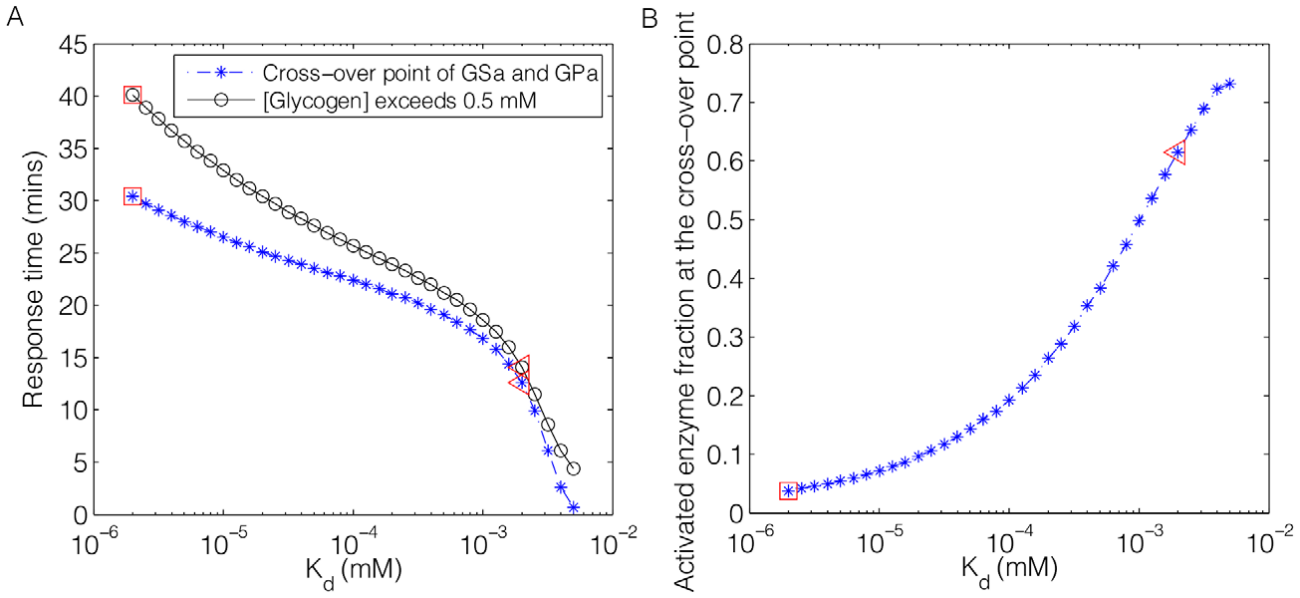
Enzymes and substrate responses over a series of 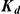
 **ranging from** 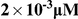 **to** 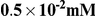 **.** A: System response time to glucose stimulus plotted against 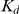 in a glycogen depleted liver. Blue: system response time defined by the cross-over point of glycogen synthase a (GSa) and glycogen phosphorylase a (GPa). Black: system response time defined by the time when liver glycogen concentration exceeds 0.5 mM. Note that the difference in system response time is about 30 mins for the lowest and highest values of 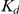 selected here under both definitions. B: The co-activated percentage of GSa and GPa at the cross-over point as a function of 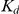. Note that this percentage represents the maximum co-active percentage of both enzymes, hence it is an indicator of the level of substrate cycling in the system. The points inside the rectangles (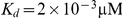) and triangles (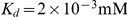) are the two values chosen in Figure 6.

We next further investigated the relationship between the system response time and the level of substrate cycling in a glycogen depleted liver. Instead of two values of 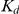 (marked by a red square (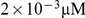) and triangle (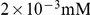) in Figure 7), we considered a range of values from 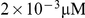 to 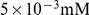. There are two different ways to define the system response time to glucose stimulus: (1) the time when the GSa and GPa curves intersect or (2) the time when glycogen concentration exceeds a threshold value. We selected a threshold value of 0.5 mM, the glycogen concentration reached at the end of the simulation (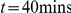) with the smallest 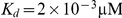. The time response curves under both definitions were shown in Figure 7A as a blue and black line respectively. The differences in the response time shown on both curves were on the order of 30 mins between the largest and smallest 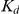. In Figure 7B, we provided the co-active percentage of GS and GP at the intersection point.

The results from this analysis provide a possible explanation as to why the biological system has different metabolic mechanisms (different 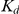) under different fasting states. In a glycogen-depleted state, it is essential to have a highly responsive system, ready for replenishing energy reserves as soon as nutrients become available. Our simulation results clearly showed that the high degree of substrate cycling occurring in the fasted state accelerated the system response in this respect by about 30 minutes, which would be physiologically significant for survival. Conversely, avoiding substrate cycling in a fed state is also desirable from an energy expenditure standpoint, as the combination of reactions involving GS, GP, glucose 1-phosphate uridylytransferase and nucleoside diphosphate kinase result in an ATP consuming reaction (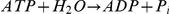).

### Comparison with experiments

**Figure 8:**
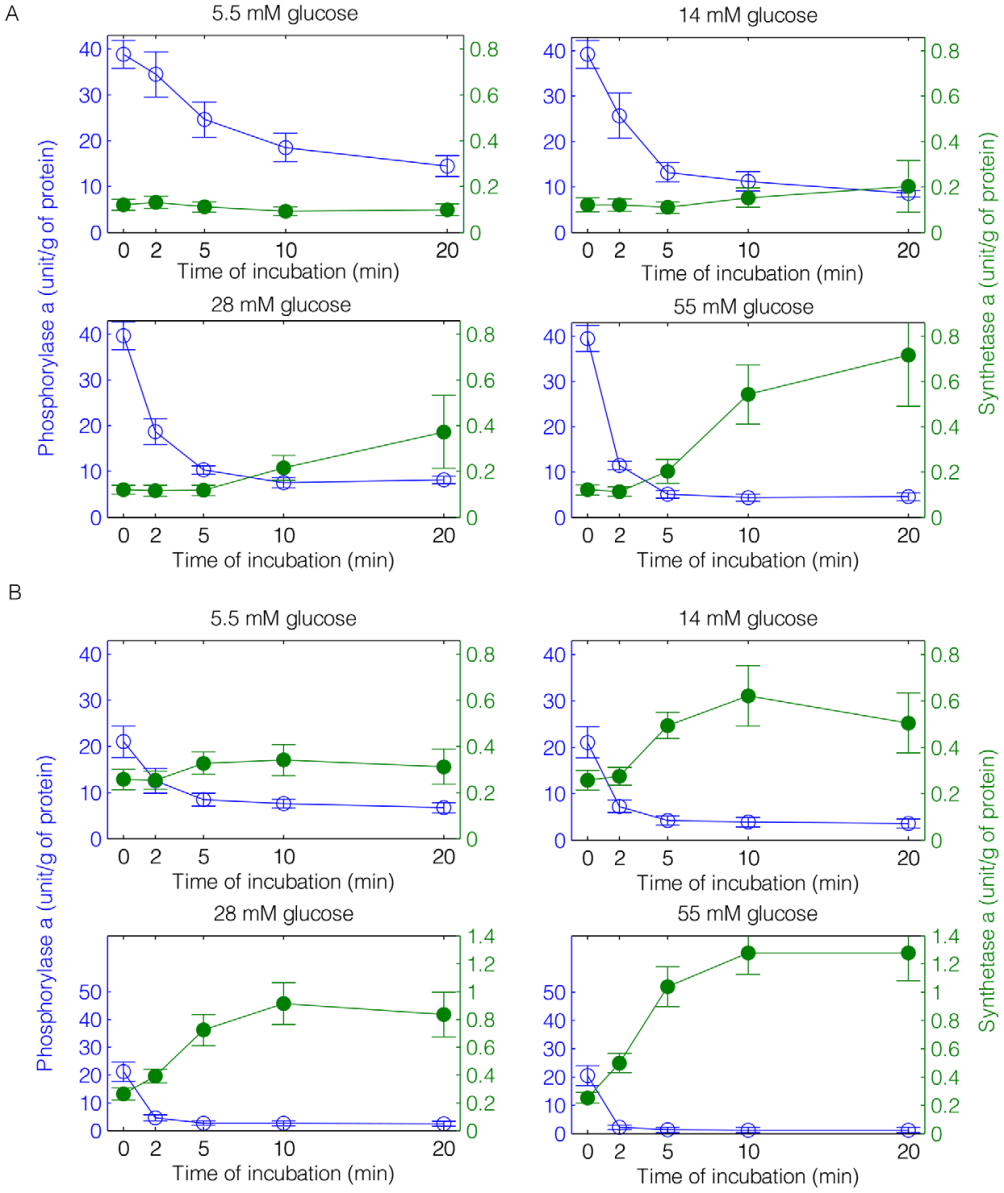
Previous experimental results by Hue et al. Glycogen phosphorylase a (GPa) and glycogen synthase a (GSa) activities in hepatocytes under fed (A) and fasted conditions (B) are redrawn from experimental results by Hue et al. [49]). From left to right, top to bottom in panel A and B: 4 increasing glucose concentrations from 5.5 to 55 mM in the incubation medium caused a sequential inactivation of glycogen phosphorylase and activation of glycogen synthase.

We have shown that the level of inhibition of GS phosphatase by GPa through the dissociation constant 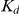, or equivalently the level of substrate cycling, determined the system response time in a glycogen depleted liver. Previous studies have shown that this inhibition is glycogen dependent [41], [42]. Watts et al. [47] reported that the GS phosphatase activity decreased in the livers of fasted, fed and gsd/gsd (liver glycogen storage disorder) mice and the addition of glycogen to homogenates of liver from starved rats reduced the glycogen synthase phosphatase activity. More recently, Armstrong et al. [48] pointed out that there are unique binding sites for GPa, PP-1 and glycogen in the hepatic glycogen-targeting subunit of protein phosphatase 1 (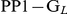), a GS phosphatase specific to liver. Therefore, it is reasonable to assume that this inhibitory regulation changes according to the liver glycogen level. We modeled this effect by using the following expression for the dissociation constant 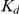:
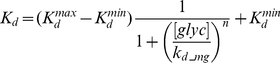
(8)where [glyc] is the liver glycogen concentration, 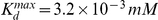, 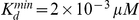, 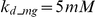 and the Hill constant 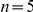. Note that the parameters were chosen to match the experimental results of [49] as shown in Figure 8.

We compared our model predictions to experimental studies that investigated GS and GP levels within fed and fasted livers in a rodent model system [49]. In this work, Hue et al. measured GP and GS activities over time in isolated hepatocytes under sequential changes to the glucose concentration (from 5.5 mM to 55 mM) in the incubation medium. Results from this study were redrawn in Figure 8.

**Figure 9:**
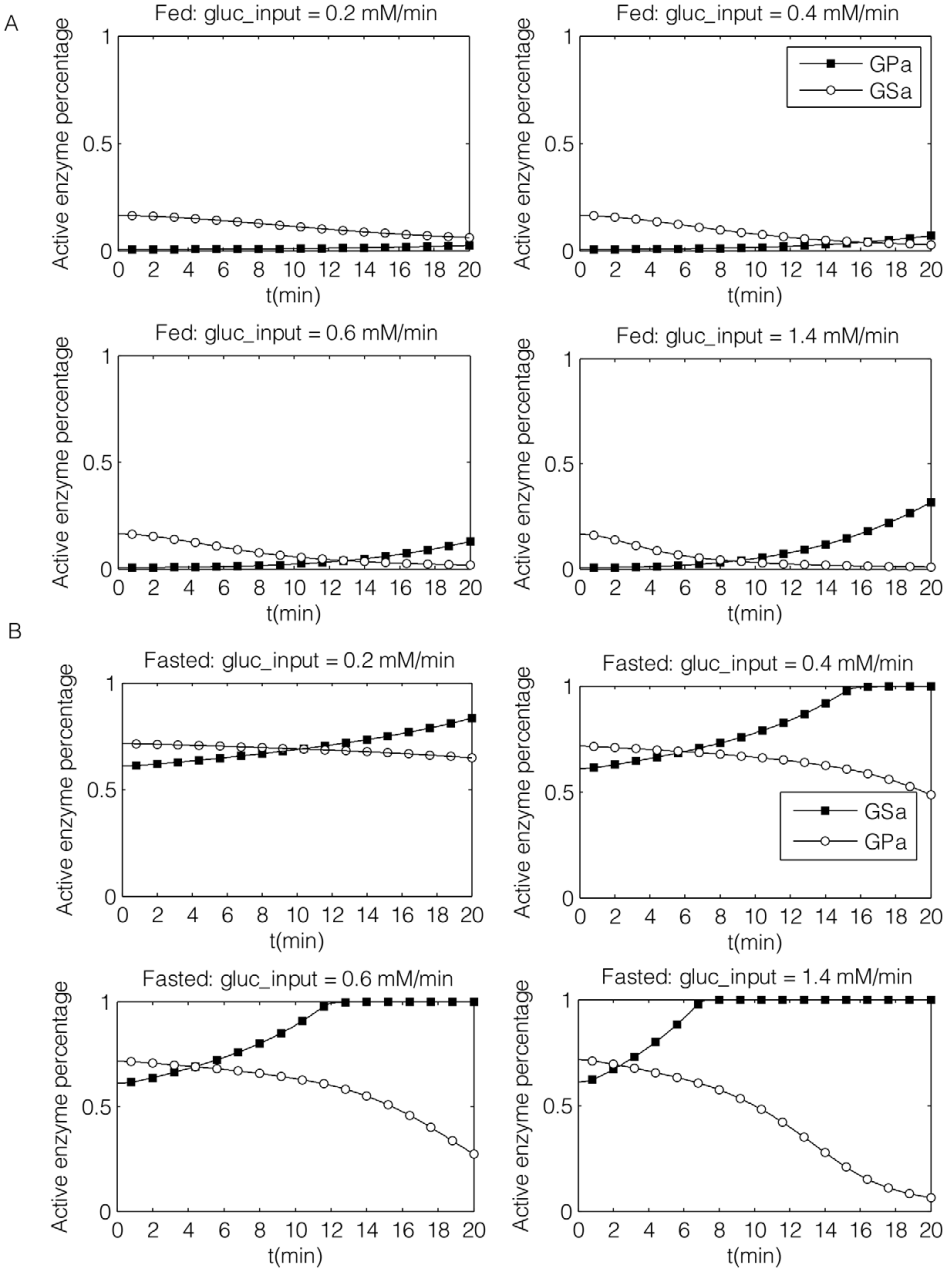
Simulation results by computational modeling (a parallel comparison to **Figure 8**). Glycogen synthase a (solid circles) and glycogen phosphorylase a (open circles) activities are plotted against time under 4 different glucose input rate in fed (A) and fasted livers (B). From left to right, top to bottom: 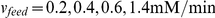. Note that the y-axis is the active to total enzyme percentage.

It is important to note that we are comparing a “whole-body” simulation with results obtained from cultured cells which are not interacting with events driven by other tissues, such as fat and muscle. However, this comparison demonstrates clear similarities between these cell culture data and our simulations with respect to responses of the glycogen regulatory circuitry to blood glucose concentrations. We started our simulation at the fed steady state and fasted the model system to two different times, 250 mins and 1200 mins, to represent fed and fasted livers respectively. In the simulation for fed livers, 250 mins fasting time was chosen to recreate a fasting environment as seen at the beginning of the experiments (Figure 8A), where GP is mostly in the active form and over 90% GS is in the inactive form [49]. Note that after 250 mins, the liver glycogen level was at about 75% of the fed steady state. In the simulation for fasted livers, 1200 mins was chosen after which only less than 1% glycogen remained. We then compared the response from both livers under 4 different glucose feeding rates (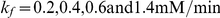), as shown in Figure 9. Since we made our observation only after glucose supply re-entered the blood stream, we shifted the simulation time forward to 250 mins and 1200 mins in the fed and fasted livers and denoted them as 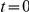.

Multiple aspects of our simulation results matched reported experimental observations of [49]. For instance, simulations and experiments showed the activation of GS to be highly suppressed by GPa in the fed state. For the lowest glucose injection rate, 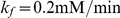, GS is not activated at all, which was also observed in experiments by Hue et al. (Figure 8A). Both the experimental and simulation results showed that the active percentage of GS was higher in the fasted than in the fed state at the end of the experiment/simulation (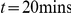). Furthermore, in both experimental studies and simulations, GS always responded more rapidly (on the order of 10–15 minutes as defined by the cross-over point of GSa and GPa) in the glycogen-depleted compared to the fed state. As the injection rate of glucose increased, the response time of GSa was shortened. Note that the glycogen concentrations from our simulation are provided in Figure S6 in Protocol S1, which also indicated a quicker response from the fasted livers. Although we can accurately capture the changes in response time under different glucose concentrations, it is clear that we have only addressed limited aspects of the relevant metabolic pathways and associated regulatory components. For instance, it is known that bioenergetics is regulated by a number of mechanisms including push-pull [50] and negative feedback, the latter being an integral component of our whole-body and glycogen-specific models. Furthermore, transcript level regulation is required to capture variations in enzyme concentration that occur under different fasting conditions. Such investigations lie outside the scope of the current model.

### Conclusions

The cells, tissues, organs, bodies and populations of all living organisms are in a constant state of sensing and response to numerous external and self-generated stimuli [51]. Feedback loops, both positive and negative, play intrinsic roles in homeostatic regulation of biological systems. Negative feedback loops underpin the majority of the balances of nature, from predator-prey relationships to biochemical networks, and are clearly subjected to evolutionary pressures [52]. Negative feedback is a common mode of control for signaling networks [53], reducing time required to reach steady states [30], providing a mechanism for reducing fluctuations in protein expression levels and pathway activity. In contrast to stabilizing activity, in the presence of sufficient time delays, negative feedback can have destabilizing effect and generate overshooting and random oscillations, rendering noise a challenging issue in the modeling of biochemical networks [54]. No attempt was made to incorporate stochasticity into the present investigations. Biological systems employ negative feedback combined with controlled time delays as a means of inducing functional oscillations. Such internally generated oscillations are responsible in large part for circadian rhythms and the cell cycle [55], which are intimately linked to the subject of feed-fasting cycles in the present work.

In this work, we have developed a physiological model that simulates selected major components of bioenergetics as outlined in [25]–[27]. The outer general bioenergetics model (outer ring of Figure 2) was created as a “test bed” [56], [57] for the glycogen circuit, which permits simulation of the glycogen regulatory circuitry in response to physiological changes that mimic the effects of fasting and feeding on whole-body energetics. As we could find no such testbed for the hepatic glycogen regulatory circuit that we were investigating, and as such circuits interact in potentially unpredictable ways with other body systems via the vascular, nervous, and other communication systems, we endeavored to build such a software platform for our investigations. Analysis of this model suggests that the glycogen circuit's context-dependent (fed or fasted) architecture allows for a significant increase in response time when the organism is in a fasted state. Suppression of substrate cycling in the fed state could provide a strategy for energy conservation leading to optimal energy storage.

The current work also provides a platform for further investigation into bioenergetic diseases such as diabetes and glycogen storage disease (GSD). Type VI and type IX GSD, representing 25–30% of the total cases, are either due to a deficiency in glycogen phosphorylase or an abnormality in the enzyme that activates it [58]. Therefore, it is crucial to understand glucose-glycogen metabolism in a whole body environment, especially the regulatory mechanisms for some of the key enzymes in these pathways such as glycogen synthase and glycogen phosphorylase. Interestingly, this work could also be of value for research into optimization of nutrition protocols for athletes or soldiers who are required to perform under stress. Glycogen supercompensation, where glycogen storage ability is increased following glycogen depletion when consuming a high carbohydrate diet, is an important issue for performance in athletes. Numerous studies have been carried out to investigate the relationship between the amount and type of carbohydrate ingestion and the maximum glycogen resynthesis rate [59]–[61]. Of related interest, a study by Roberts et al. [62] demonstrated that metabolism of simple sugars leads to a higher glycogen resynthesis rate than that generated through the metabolism of complex carbohydrates. Under the current computational model platform, these observations could be further investigated in a continuous parameter space, and an optimal nutrition plan for these individuals might be predicted by taking into account energy flows. Computational models, such as the one developed here, could assist in the design of nutrition plans for athletes and individuals suffering from bioenergetic challenges, including diabetes.

## Materials and Methods

One of the goals of our metabolic model was to capture key features of the dynamics of internal energy sources, from fed through fasted states, to include blood glucose, liver glycogen, FFAs, and ketone bodies, regulated by plasma glucagon and insulin. The dynamics of these substrates and enzymes are described in [25]–[27], while the whole body energetics have been reviewed in [26], [28], [29]. A summary of these time events is given in [63]. Such a simulation would then provide a dynamic framework within which to test the behavior of the underlying control circuitry, as for glycogen in the present study. When the physiological system enters the fasting state, blood glucose concentration drops, flipping a reciprocal switch with respect to plasma insulin and glucagon concentrations [28], [36]. cAMP then responds and transmits a signal to the glycogen circuitry to regulate the activities of GP and GS [7]. As a result, hepatic glycogen is being depleted as it is catabolized to maintain blood glucose levels within the physiological range needed for survival. The level of plasma free fatty acids and ketone bodies also rise to provide alternative metabolic fuels. A diagram of the concentrations of selected metabolites with respect to time after fasting commences is available in Figure 3. Except for the similar characteristic behaviors described previously in [25]–[29], our model is also able to capture the damped oscillations at the beginning of a new local stable state.

### Metabolic pathways

Here we give a brief overview of the four major compartments in our liver-centered physiological model, as shown in Figure 2. For a detailed description of these pathways, model equations and parameters, please refer to Protocol S1. A detailed parameter-based sensitivity analysis has also been conducted and results revealed that blood glucose is not sensitive to 10-fold changes in the parameters that describe the activity of each enzyme. The results are provided in Table S8–S10 in Protocol S1.

#### Liver

As a center of bioenergetic regulation, the liver is able to convert glucose that is surplus to immediate energy demands into a short term energy storage form, glycogen, and utilize it to maintain blood glucose homeostasis during the early stages of fasting. Besides its regulatory functions in glycogenesis and glycogenolysis, the liver is also capable of processing amino acids and free fatty acids from muscle and adipose tissue, respectively, to stabilize the blood glucose level when hepatic glycogen is depleted. Recognizing its irreplaceable role in metabolism, the liver is modeled extensively within our physiological network. The eight simplified pathways in the liver include glycolysis, gluconeogenesis, glycogenesis, glycogenolysis, TCA cycle, lipogenesis, lipolysis and ketogenesis.

#### Adipose tissue

As mentioned above, glycogen is a effective short term energy reserve because it can be catabolized quickly to satisfy a urgent need for glucose. In terms of long term energy storage, however, it is not as effective as triglycerides, which is a much more compact energy storage device which is largely contained within adipose tissues. Since our model is focused on the liver's role in metabolism, a brief sketch of the metabolic interactions between this organ and those in the fat and muscle tissue are included in our simulations. An important role of fat is that glucose is taken up by fat in an insulin-dependent manner and is stored as triacylglycerol within fat as an energy depot that is available to drive gluconeogenesis when blood glucose levels drop and glucose generation is needed. The conversion of glucose into and free fatty acids in fat tissue are described in Protocol S1. The only metabolites modeled for fat tissue are glucose-6-phosphate, acyl-CoA, triacylglycerol and free fatty acids.

#### Muscle

Muscle is another major site for glycogen storage. However, muscle glycogen cannot contribute directly to plasma glucose since muscle lacks glucose-6-phosphatase, an important enzyme in the gluconeogenesis pathway. Instead, the end product of glycolysis, pyruvate, can either form lactate or alanine (our representative for amino acids and proteins), to be transported to the liver. Alanine is a direct substrate for gluconeogenesis in liver while lactate assists in the maintenance blood glucose level through the Cori cycle, in which lactate is used in gluconeogenesis in liver. In our model, muscle is modeled as a sink term for metabolic fuels such as glucose and ketone bodies and also an alternative energy source to provide lactate and alanine. The metabolites modeled for muscle are glycogen, glucose-6-phosphate, pyruvate, lactate, alanine and ketone bodies.

#### Blood

For simplification, all the metabolic processes (except for degradation) are ignored in the blood. It only serves as a transport system, conveying nutrients between the major organs simulated by the model.

All the transport processes described above are shown in Figure S4 in Protocol S1 and a more detailed description is also provided there.

## Supporting Information

Protocol S1 Detailed model equations and supplemental figures.
(DOC)

Protocol S2 MATLAB Package.
(RAR)

Protocol S3 Readme file for the MATLAB package.
(DOCX)

Protocol S4 SBML version of the MATLAB package.
(XML)
