# Supplementary material for: A Whole-Body Model for Glycogen Regulation Reveals a Critical Role for Substrate Cycling in Maintaining Blood Glucose Homeostasis
Source: PLoS Comput Biol. 2011 Dec 1;7(12):e1002272. doi: 10.1371/journal.pcbi.1002272 (PMC3233304; doi:10.1371/journal.pcbi.1002272)
Supplement: Protocol S1 — Detailed model equations and supplemental figures. (DOC) [file pcbi.1002272.s001.doc]

# Supplementary Material – Model details

for

**A Whole-body Model for Glycogen Regulation Reveals A Critical Role for Substrate Cycling in Maintaining Blood Glucose**

Ke Xu^1,2^, Kevin Morgan^3^, Abby Todd Gehris^4^, Timothy C Elston^2,*^, Shawn M Gomez^1,2,5,*^

Addresses:

1 Department of Biomedical Engineering, University of North Carolina School of

Medicine, Chapel Hill, North Carolina 27599, USA

2 Department of Pharmacology, University of North Carolina School of Medicine, Chapel Hill, North Carolina 27599, USA

3 Old Dogs in Training LLC, Carrboro, North Carolina 27510, USA

4 Department of Mathematics, Broome Community College, Binghamton, New York

13902, USA

5 Department of Computer Science, University of North Carolina at Chapel Hill, Chapel

Hill, North Carolina, USA

∗ E-mail: telston@amath.unc.edu, smgomez@unc.edu

**Part I: Model Equations ([1])**

**1. Rates and Equations for the Whole-body Physiological Model**

**LIVER RATES:**

$$v_{L1}\text{ }=\text{ }\frac{k_{L1}\text{ }[gluc]}{k_{mL1}\text{ }+\text{ }[gluc]}\text{ }\left(1\text{ }+\text{ }\frac{[B\_ins]^{ep1}}{k_{Dins}^{ep1}\text{ }+\text{ }[B\_ins]^{ep1}}\right)$$

$$v_{L2}\text{ }=\text{ }\frac{k_{L2}\text{ }[GSa]\text{ }[g6p]}{k_{mL2}\text{ }+\text{ }[g6p]}$$

$$v_{L5}\text{ }=\text{ }\frac{k_{L5}\text{ }[pyr]}{k_{mL5}\text{ }+\text{ }[pyr]}$$

$$v_{-L5}\text{ }=\text{ }\frac{k_{-L5}\text{ }[lac]}{k_{m-L5}\text{ }+\text{ }[lac]}$$

$$v_{L6}\text{ }=\text{ }\frac{k_{L6}\text{ }[pyr]}{k_{mL6}\text{ }+\text{ }[pyr]}\text{ }\left(1\text{ }+\text{ }\frac{p_{2}\text{ }[acet\_m]}{k_{p2}\text{ }+\text{ }[acet\_m]}\right)\text{ }\left(1\text{ }+\text{ }\frac{[B\_glucgn]^{ep4}}{k_{Dglucgn}^{ep4}\text{ }+\text{ }[B\_glucgn]^{ep4}}\right)$$

$$v_{L7}\text{ }=\text{ }\frac{k_{L7}\text{ }[pyr]}{k_{mL7}\text{ }+\text{ }[pyr]}\text{ }\frac{k_{i8}}{k_{i8}\text{ }+\text{ }[acet\_m]}$$

$$v_{L21}\text{ }=\text{ }k_{L21}\text{ }\frac{[pyr]}{k_{mL21p}\text{ }+\text{ }[pyr]}\text{ }\frac{[glutamate]}{k_{mL21g}\text{ }+\text{ }[glutamate]}\text{ }\frac{k_{Dins}^{en3}}{k_{Dins}^{en3}\text{ }+\text{ }[B\_ins]^{en3}}$$

$$v_{-L21}\text{ }=\text{ }k_{-L21}\text{ }\frac{[alan]}{k_{m-L21a}\text{ }+\text{ }[alan]}\text{ }\frac{[aK]}{k_{m-L21k}\text{ }+\text{ }[aK]}\text{ }[acet\_m]\text{ }\frac{k_{Dins}^{en3}}{k_{Dins}^{en3}\text{ }+\text{ }[B\_ins]^{en3}}$$

**BLOOD RATES (Transport and Decay):**

$$v_{tL3}\text{ }=\text{ }k_{tL3}\text{ }[ket]$$

$$v_{tL5}\text{ }=\text{ }k_{tL5}\text{ }[B\_ffa]$$

$$v_{tL6}\text{ }=\text{ }k_{tL6}\text{ }[B\_alan]\text{ }\frac{k_{Dins}^{en10}}{k_{Dins}^{en10}\text{ }+\text{ }[B\_ins]^{en10}}$$

$$v_{tF1}\text{ }=\text{ }k_{tF1}\text{ }[B\_gluc]\text{ }\left(1\text{ }+\text{ }\frac{[B\_ins]^{ep12}}{k_{Dins}^{ep12}\text{ }+\text{ }[B\_ins]^{ep12}}\right)$$

$$v_{tF3}\text{ }=\text{ }k_{tF3}\text{ }[F\_ffa]^{8}\text{ }\frac{k_{Dins2}^{en9}}{k_{Dins2}^{en9}\text{ }+\text{ }[B\_ins]^{en9}}$$

$$v_{tS1}\text{ }=\text{ }k_{tS1}\text{ }[B\_gluc]\text{ }\left(1\text{ }+\text{ }\frac{[B\_ins]^{ep13}}{k_{Dins}^{ep13}\text{ }+\text{ }[B\_ins]^{ep13}}\right)$$

$$v_{tS2}\text{ }=\text{ }k_{tS2}\text{ }[B\_ket]$$

**FAT RATES:**

**MUSCLE RATES:**

**HORMONAL GROWTH RATES:**

**MODEL EQUATIONS:**

$$\frac{d[acet\_m]}{dt}\text{ }=\text{ }v_{L7}\text{ }-\text{ }v_{L8}\text{ }+\text{ }8\text{ }v_{L18}\text{ }-\text{ }2\text{ }v_{L19}$$

$$\frac{d[citrate]}{dt}\text{ }=\text{ }v_{L8}\text{ }-\text{ }v_{L9}\text{ }-\text{ }v_{L14}$$

$$\frac{d[aK]}{dt}\text{ }=\text{ }v_{L9}\text{ }-\text{ }v_{L10}\text{ }+\text{ }v_{L21}\text{ }-\text{ }v_{-L21}$$

$$\frac{d[malate]}{dt}\text{ }=\text{ }v_{L10}\text{ }+\text{ }v_{L11}\text{ }-\text{ }v_{-L11}\text{ }-\text{ }v_{L12}$$

$$\frac{d[oa\_c]}{dt}\text{ }=\text{ }v_{L12}\text{ }-\text{ }v_{L13}\text{ }+\text{ }v_{L14}$$

$$\frac{d[acet\_c]}{dt}\text{ }=\text{ }v_{L14}\text{ }-\text{ }v_{L15}\text{ }-\text{ }v_{L16}$$

$$\frac{d[malonyl]}{dt}\text{ }=\text{ }v_{L15}\text{ }-\text{ }7\text{ }v_{L16}$$

$$\frac{d[palm]}{dt}\text{ }=\text{ }v_{L16}\text{ }-\text{ }v_{L17}\text{ }+\text{ }v_{tL5}$$

$$\frac{d[palmCoA]}{dt}\text{ }=\text{ }v_{L17}\text{ }-\text{ }v_{L18}$$

$$\frac{d[ket]}{dt}\text{ }=\text{ }v_{L19}\text{ }-\text{ }v_{tL3}$$

$$\frac{d[alan]}{dt}\text{ }=\text{ }v_{tL6}\text{ }+\text{ }v_{L21}\text{ }-\text{ }v_{-L21}\text{ }-\text{ }v_{L20}$$

$$\frac{d[B\_lac]}{dt}\text{ }=\text{ }v_{tS3}\text{ }-\text{ }v_{tL2}\text{ }-\text{ }v_{d\_Blac}$$

$$\frac{d[B\_ket]}{dt}\text{ }=\text{ }v_{tL3}\text{ }-\text{ }v_{tS2}\text{ }-\text{ }v_{d\_Bket}$$

$$\frac{d[B\_alan]}{dt}\text{ }=\text{ }v_{tS4}\text{ }-\text{ }v_{tL6}\text{ }-\text{ }v_{d\_Balan}$$

$$\frac{d[F\_g6p]}{dt}\text{ }=\text{ }v_{tF1}\text{ }-\text{ }v_{F1}$$

$$\frac{d[F\_acyl]}{dt}\text{ }=\text{ }v_{F1}\text{ }-\text{ }v_{F3}\text{ }+\text{ }v_{F5}$$

$$\frac{d[F\_TG]}{dt}\text{ }=\text{ }v_{F3}\text{ }-\text{ }v_{F4}$$

$$\frac{d[F\_ffa]}{dt}\text{ }=\text{ }0$$

**2 Rates and Equations for Glycogen Regulatory Circuit Model**

**GLYCOGEN REGULATORY CIRCUIT:**

**Part II : Tables**

**1. Parameter Values ([1])**

Note that where literature was available, parameters were selected to meet published criteria (Table S.1-S.7). Where it was not, the parameters were chosen to be within a physiologically relevant range and at the same time minimize the discrepancy from the experiments by Hue et al.

**Table S1 Parameter Values for Liver Reactions**

| *Reaction* | *Parameters* |  | *Reaction* | *Parameters* |
| --- | --- | --- | --- | --- |
| *v_L_*_1_ | *k_L_*_1_ = 3 mM*min^-1^  *k_mL_*_1_ = 7.7 mM [2]  *ep*1 = 10 |  | *v_L_*_6_ | *k_L_*_6_ = 1 mM*min^-1^  *k_mL_*_6_ = 0.22 mM[3]  *p*_2_ = 1  *k_p_*_2_ = 2 mM  *ep*4 = 10 |
| *v_-L_*_1_ | *k_-L_*_1_ = 4 mM*min^-1^  *k_m-L_*_1_ = 1.3 mM[4]  *ep*9 = 10 |  | *v_L_*_7_ | *k_L_*_7_ = 1 mM*min^-1^  *k_mL_*_7_ = 0.0204 mM [5]  *k_i_*_8_ = 2 mM |
| *v_L_*_2_ | *k_L_*_2_ = 200 min^-1^  *k_mL_*_2_ = .57 mM[6] |  | *v_L_*_8_ | *k_L_*_8_ = 0.1 mM^-1^*min^-1^  *k_i_*_4_ = 3 mM |
| *v_-L_*_2_ | *k*-*_L_*_2_ = 20 min^-1^  *k_m_*-*_L_*_2_ = 1.4 mM[7] |  | *v_L_*_9_ | *k_L_*_9_ = 0.1 min^-1^ |
| *v_L_*_3_ | *k_L_*_3_ = 0.1 mM*min^-1^  *k_mL_*_3_ = .01 mM[8]  *ep*2 = 10  en1 = 10 |  | *v_L_*_10_ | *k_L_*_10_ = 0.1 min^-1^ |
| *v*-*_L_*_3_ | *k*-*_L_*_3_ = .1 mM*min^-1^  *k_m_*-*_L_*_3_ = 0.0034 mM [9]  *ep*8 = 10  *en*6 = 10 |  | *v_L_*_11_ | *k_L_*_11_ = 0.6 min^-1^  *ep*5 = 10 |
| *v_L_*_4_ | *k_L_*_4_ = 2 mM*min^-1^,  *k_mL_*_4_ = 0.18 mM [10]  *k_i_*_13_ = 2 mM,  *ep*3 = 10,  *en*2 = 10 |  | *v_-L_*_11_ | *k*-*_L_*_11_ = 0.01 min^-1^ |
| *v_L_*_5_ | *k_L_*_5_ = 0  *k_mL_*_5_ = .03mM [11] |  | *v_L_*_12_ | *k_L_*_12_ = 0.6 min^-1^  *ep*6 = 10  *en*4 = 10 |
| *v*-*_L_*_5_ | *k*-*_L_*_5_ = 0.1 mM*min^-1^  *k_m_*-*_L_*_5_ = 0.8 mM[12] |  | *v_L_*_13_ | *k_L_*_13_ = 0.5 min^-1^  *ep*7 = 10  *en*5 = 10 |
| *v_L_*_14_ | *k_L_*_14_ = 0.01 min^-1^  *ep*10 = 10  *en*7 = 10 |  | *v_L_*_19_ | *k_L_*_19_ = 0.01 mM^-1^*min^-1^  *ep*11 = 10  *en*8 = 10 |
| *v_L_*_15_ | *k_L_*_15_ = 0.01 min^-1^  *p*_1_ = 1  *k_p_*_1_ = 0.5 mM  *k_i_*_5_ = 1 mM |  | *v_L_*_20_ | *k_L_*_20_ = 0.2 min^-1^ |
| *v_L_*_16_ | *k_L_*_16_ = 0.01 mM^-7^*min^-1^ |  | *v_L_*_21_ | *k_L_*_21_ = 0.001 mM*min^-1^  *en*3 = 10  *k_mL_*_21p_ = 0.4 mM [13, 14]  *k_mL_*_21g_ = 4.3 mM[123 15] |
| *v_L_*_17_ | *k_L_*_17_ = 0.01 mM*min^-1^  *k_i_*_1_ = 0.1 mM |  | *v*-*_L_*_21_ | *k*-*_L_*_21_ = 0.2 min^-1^  *en*3 = 10  *k_m-L_*_21a_ = 21 mM[16]  *k_m-L_*_21k_ = 0.22 mM[16] |
| *v_L_*_18_ | *k_L_*_18_ = 0.01 min^-1^  *k_i_*_2_ = 1 mM |  | *v_L_*_22_ | *k_L_*_22_ = 0.01 min^-1^ |

**Table S2 Parameter Values for Blood Reactions (Transport, Decay and Feed)**

| *Reaction* | *Parameters* |  | *Reaction* | *Parameters* |
| --- | --- | --- | --- | --- |
|  |  |  |  |  |
| *v_tL_*_1_ | *k_tL_*_1_ = 100 min^-1^ |  | *v_tF_*_1_ | *k_tF_*_1_ = 0.01 min^-1^  *ep*12 = 10 |
| *v_tL_*_2_ | *k_tL_*_2_ = 0.1 min^-1^ |  | *v_tF_*_3_ | *k_tF_*_3_ = 0.01 mM^-7^*min^-1^  *en*9 = 20 |
| *v_tL_*_3_ | *k_tL_*_3_ = 0.1 min^-1^ |  | *v_tS_*_1_ | *k_tS_*_1_ = 0.01 min^-1^  *ep*13 = 10 |
| *v_tL_*_4_ | *k_tL_*_4_ = 0.1 min^-1^ |  | *v_tS_*_2_ | *k_tS_*_2_ = 1 min^-1^ |
| *v_tL_*_5_ | *k_tL_*_5_ = 0.1 min^-1^ |  | *v_tS_*_3_ | *k_tS_*_3_ = 0.01 min^-1^ |
| *v_tL6_* | *k_tL_*_6_ = 1 min^-1^  *en*10 = 10 |  | *v_tS_*_4_ | *k_tS_*_4_ = 3 min^-1^  *en*11 = 10 |
|  |  |  |  |  |
| *vd_Bgluc* | *kd_Bgluc* = 0.015 min^-1^ |  | *vd_Blac* | *kd_Blac* = 0.01 min^-1^ |
| *vd_Bins* | *kd_Bins* = 0.015 min^-1^ |  | *vd_Bket* | *kd_Bket* = 0.01 min^-1^ |
| *vd_Bglucgn* | *kd_Bglucgn* = 0.015 min^-1^ |  | *vd_Balan* | *kd_Balan* = 0.01 min^-1^ |
| *vd_Bffa* | *kd_Bffa* = 0.015 min^-1^ |  | *v_feed_* | *k_feed_* = 0.5 mM*min^-1^ |

**Table S3 Parameter Values for Fat Reactions**

| *Reaction* | *Parameters* |  | *Reaction* | *Parameters* |
| --- | --- | --- | --- | --- |
|  |  |  |  |  |
| *v_F_*_1_ | *k_F_*_1_ = 0.1 min^-1^  *ep*14 = 10 |  | *v_F_*_4_ | *k_F_*_4_ = 0.1 min^-1^  *en*12 = 10 |
| *v_F_*_3_ | *k_F_*_3_ = 0.2 min^-1^  *ep*15 = 10 |  | *v_F_*_5_ | *k_F_*_5_ = 0.1 mM^-2^*min^-1^ |

**Table S4 Parameter Values for Muscle Reactions**

| *Reaction* | *Parameters* |  | *Reaction* | *Parameters* |
| --- | --- | --- | --- | --- |
|  |  |  |  |  |
| *v_S_*_1_ | *k_S_*_1_ = 0 |  | *v_S_*_4_ | *k_S_*_4_ = 0.07 min^-1^ |
| *v*-*_S_*_1_ | *k*-*_S_*_1_ = 0 |  | *v*-*_S_*_4_ | *k*-*_S_*_4_ = 0 |
| *v_S_*_2_ | *k_S_*_2_ = 0.02 min^-1^ |  | *v_S_*_5_ | *k_S_*_5_ = 0.5 min^-1^ |
| *v_S_*_3_ | *k_S_*_3_ = 0.01 min^-1^ |  | *vS_dket* | *kS_dket* = 0.01 min^-1^ |
| *v*-*_S_*_3_ | *k*-*_S_*_3_ = 0.01 min^-1^ |  |  |  |

**Table S5 Parameter Values for Glycogen Regulatory Circuit Reactions**

| *Reaction* | *Parameters* |  | *Reaction* | *Parameters* |
| --- | --- | --- | --- | --- |
| *v_g_*_3_ | *k_g_*_3_ = 1200 min^-1^ [17]  *k_mg_*_3_ = 0.0004 mM [17] |  | *v_g_*_6_ | *k_g_*_6_ = 300 min^-1^ [17, 18]  *k_mg_*_6_ = 0.005 mM [17, 19]  *s*_2_ = .001 [17]  *k_gi_* = 10 mM [17] |
| *v_g_*_4_ | *k_g_*_4_ = 300 min^-1^ [17]  *k_mg_*_4_ = 0.0011 mM [17, 20] |  | *v_g_*_7_ | *k_g_*_7_ = 1200 min^-1^ [17]  *k_mg_*_7_ = 0.015 mM[17] |
| *v_g_*_5_ | *k_g_*_5_ = 1200 min^-1^[17, 21]  *k_mg_*_5_ = 0.01 mM[17, 22] |  | *v_g_*_8_ | *k_g_*_8_ = 300 min^-1^  *k_mg_*_8_ = 0.00012 mM [17, 23] |
| *v_g_*_5_, *v_g_*_7_, *v_g_*_8_ | *s*_1_ = 100 [17]  *k_g_*_2_ = 0.5 mM [17] |  |  |  |

**Table S6 Additional Parameter Values for Glycogen Regulatory Circuit in Isolation ([17, 24-26])**

| *Parameters* |  | *Parameters* |  | *Parameters* |
| --- | --- | --- | --- | --- |
| *capkt* = 0.00025 mM |  | *PP2A* = 0.000025 mM |  | *g6pt* = 0.7 mM |
| *It* = 0.0018 mM |  | *k11* = 0.000043 mM |  | *kgi* = 10 mM |
| *kt* = 0.0025 mM |  | *k22* = 0.0007 mM |  | *s_1_* = 100 |
| *pt* = 0.07 mM |  | *ki* = 0.1 mM |  | *kg2* = 0.5 mM |
| *st* = 0.003 mM |  | *campt* = 0.01 mM |  | *s_2_* = 0.001 |
| *PP1* = 0.00025 mM |  | *kg* = 349.5 mM |  |  |

**Table S7 Parameter Values Pertaining to Non-Metabolite Regulators (Insulin, Glucagon and cAMP)**

| *Reaction* | *Parameters* |  | *Reaction* | *Parameters* |
| --- | --- | --- | --- | --- |
| *v_L_*_1_, *v_L_*_3_, *v_L_*_4_, *v_L_*_12_, *v_L_*_13_, *v_L_*_14_, *v_L_*_19_, *v_L_*_21_, *v_-L_*_21_, *v_tL_*_6_, *v_tF_*_1_, *v_tS_*_1_, *v_tS_*_4_, *v_F_*_1_, *v_F_*_3_, *v_F_*_4_ | *k_Dins_* = 1 * 10^-6^ mM |  | *v_glucgn_* | *k_glucgn_* = 2 * 10^-9^ mM*min^-1^ |
| *v_-L_*_3_, *v_L_*_20_, *v_tF_*_3_ | *k_Dins2_* = 0.75 * 10^-6^ mM |  | *v_c_*_1_ | *k_c_*_1_ = 1 mM*min^-1^  *k_cm_*_1_ = 4 * 10^-8^ mM |
| *v_-L_*_1_, *v_L_*_3_, *v_-L_*_3_, *v_L_*_4_, *v_L_*_6_, *v_L_*_11_, *v_L_*_12_, *v_L_*_13_, *v_L_*_14_ | *k_Dglucgn_* = 4 * 10^-8^ mM |  | *v_c_*_2_ | *k_c_*_2_ = 1 * 10^-5.5^ mM*min^-1^  *k_cm_*_2_ = 1 * 10^-6^ mM |
| *v_L_*_19_ | *k_DcAMP_* = 1 * 10^-5.5^ mM |  | *v_gc_*_1_ | *k_gc_*_1_ = 6 * 10^10^ mM^-2^ min^-1^  *k_-gc_*_1_ = 1.3953 * 10^15^ mM^-1^ min^-1^ |
| *v_ins_* | *k_ins_* = 7 * 10^-4^ mM*min^-1^ |  | *v_gc_*_2_ | *k_gc_*_2_ = 6 * 10^10^ mM^-2^ min^-1^  *k_-gc_*_2_ = 8.5714 * 10^13^ mM^-1^ min^-1^ |
| *v_Igluc_* | *k*_1_*_ins_* = 6 * 10^-4^ mM*min^-1^  *k_mins_* = 8 mM  *ni* = 10 |  | *v_Igluc_*, *v_c_*_1_ | *ni* = 10 |
| *v_Ggluc_* | *k*_1_*_glucgn_* = 5 * 10^-9^ mM*min^-1^  *k_mGlgn_* = 8 mM  *ng* = 10 |  | *v_Ggluc_*, *v_c_*_2_ | *ng* = 10 |

**2. Parameter Sensitivity**

Sensitivity analysis is carried out for each reaction parameters, as listed before. These parameters are varied by 10 fold (up and down) to the baseline values. The sensitivity constant C is defined as the fold change in blood glucose concentration to the fold change of parameter when system reaches the fed steady state with a constant glucose input. For example, *k_L_*_1_ can be varied between [0.3, 10.17], causing a change in blood glucose from 8.48 mM to 7.10 mM.

**Table S8 Blood Glucose Sensitivity Due To 10-fold Changes In Parameter Values In Liver**

| *Reaction* | *Parameters*  *(Baseline values)* |  |  |
| --- | --- | --- | --- |
| *v_L_*_1_ | *k_L_*_1_ = 3  *k_mL_*_1_ = 7.7 [2] | 5.93 e-03  1.83 e-03 | 5.06 e-02  2.63 e-02 |
| *v_-L_*_1_ | *k_-L_*_1_ = 4  *k_m-L_*_1_ = 1.3 [4] | 5.19 e-03  2.17 e-03 | 4.83 e-02  3.59 e-02 |
| *v_L_*_2_ | *k_L_*_2_ = 200  *k_mL_*_2_ = .57 [6] | 8.58 e-04  4.16e-04 | 1.77 e-02  8.76 e-03 |
| *v_-L_*_2_ | *k*-*_L_*_2_ = 20  *k_m_*-*_L_*_2_ = 1.4 [7] | 1.10 e-04  8.55e-08 | 2.36 e-03  1.84e-06 |
| *v_L_*_3_ | *k_L_*_3_ = 0.1  *k_mL_*_3_ = .01 [8] | 5.07 e-04  7.74e-06 | 3.20 e-03  1.66 e-04 |
| *v*-*_L_*_3_ | *k*-*_L_*_3_ = .1  *k_m_*-*_L_*_3_ = 0.0034 [9] | 6.97e-05  6.36e-06 | 1.49 e-03  1.37 e-04 |
| *v_L_*_4_ | *k_L_*_4_ = 2,  *k_mL_*_4_ = 0.18 [10]  *k_i_*_13_ = 2, | 3.51e-05  6.36e-06  8.46e-06 | 2.76 e-04  1.37 e-04  8.17e-05 |
| *v_L_*_5_ | *k_L_*_5_ = 0  *k_mL_*_5_ = .03 [11] | Reaction set to 0 | Reaction set to 0 |
| *v*-*_L_*_5_ | *k*-*_L_*_5_ = 0.1  *k_m_*-*_L_*_5_ = 0.8 [12] | 9.28e-09  3.02e-13 | 1.69e-07  6.48e-12 |
| *v_L_*_6_ | *k_L_*_6_ = 1  *k_mL_*_6_ = 0.22 [3]  *p*_2_ = 1  *k_p_*_2_ = 2 | 3.97e-06  3.35e-06  1.10e-06  3.61e-07 | 5.54e-05  5.29e-05  2.36e-05  7.76e-06 |
| *v_L_*_7_ | *k_L_*_7_ = 1  *k_mL_*_7_ = 0.0204 [5]  *k_i_*_8_ = 2 | 9.06e-06  3.35e-06  4.06e-06 | 7.13e-05  5.29e-05  4.35e-05 |
| *v_L_*_8_ | *k_L_*_8_ = 0.1  *k_i_*_4_ = 3 | 1.02e-06  4.02e-07 | 2.20e-05  8.65e-06 |
| *v_L_*_9_ | *k_L_*_9_ = 0.1 | 1.51e-07 | 3.25e-06 |
| *v_L_*_10_ | *k_L_*_10_ = 0.1 | 4.02e-07 | 8.64e-06 |
| *v_L_*_11_ | *k_L_*_11_ = 0.6 | 1.02e-06 | 2.20e-05 |
| *v_-L_*_11_ | *k*-*_L_*_11_ = 0.01 | 5.17e-08 | 1.11e-06 |
| *v_L_*_12_ | *k_L_*_12_ = 0.6 | 5.17e-08 | 1.11e-06 |
| *v_L_*_13_ | *k_L_*_13_ = 0.5 | 4.42e-13 | 9.49e-12 |
| *v_L_*_14_ | *k_L_*_14_ = 0.01 | 1.48e-07 | 3.19e-06 |
| *v_L_*_15_ | *k_L_*_15_ = 0.01  *p*_1_ = 1  *k_p_*_1_ = 0.5  *k_i_*_5_ = 1 | 2.70e-08  7.43e-09  3.16e-09  1.53e-08 | 5.79e-07  1.60e-07  6.80e-08  3.29e-07 |
| *v_L_*_16_ | *k_L_*_16_ = 0.01 | 2.70e-08 | 5.79e-07 |
| *v_L_*_17_ | *k_L_*_17_ = 0.01  *k_i_*_1_ = 0.1 | 1.062e-08  7.20e-09 | 2.28e-07  1.55e-07 |
| *v_L_*_18_ | *k_L_*_18_ = 0.01  *k_i_*_2_ = 1 | 3.92e-07  2.18e-07 | 8.43e-06  4.68e-06 |
| *v_L_*_19_ | *k_L_*_19_ = 0.01 | 4.675e-07 | 1.01e-05 |
| *v_L_*_20_ | *k_L_*_20_ = 0.2 | 6.63e-06 | 6.86e-05 |
| *v_L_*_21_ | *k_L_*_21_ = 0.001  *k_mL_*_21p_ = 0.4 [13, 14]  *k_mL_*_21g_ = 4.3 [123 15] | 3.33e-10  2.74e-10  1.13e-10 | 7.17e-09  5.89e-09  2.44e-09 |
| *v*-*_L_*_21_ | *k*-*_L_*_21_ = 0.2  *k_m-L_*_21a_ = 21 [16]  *k_m-L_*_21k_ = 0.22 [16] | 4.45e-07  4.01e-07  4.02e-07 | 9.57e-06  8.61e-06  8.64e-06 |
| *v_L_*_22_ | *k_L_*_22_ = 0.01 | 1.13e-10 | 2.445e-09 |

**Table S9 Blood Glucose Sensitivity Due To 10-fold Changes In Parameter Values** In Fat

| *Reaction* | *Parameters*  *(Baseline values)* |  |  |
| --- | --- | --- | --- |
| *V_F_*_1_ | *k_F_*_1_ = 0.1 min^-1^ | 2.33e-13 | 5.01e-12 |
| *V_F_*_3_ | *k_F_*_3_ = 0.2 min^-1^ | 2.32e-13 | 4.99e-12 |
| *V_F4_* | *k_F_*_4_ = 0.1 min^-1^ | 2.32e-13 | 4.994e-12 |
| *V_F5_* | *k_F_*_5_ = 0.1 mM^-2^*min^-1^ | 2.43e-13 | 5.23e-12 |

**Table S10 Blood Glucose Sensitivity Due To 10-fold Changes In Parameter Values In Muscle**

| *Reaction* | *Parameters*  *(Baseline values)* |  |  |
| --- | --- | --- | --- |
| *v_S_*_2_ | *k_S_*_2_ = 0.02 min^-1^ | 4.00e-13 | 8.59e-12 |
| *v_S_*_3_ | *k_S_*_3_ = 0.01 min^-1^ | 5.44e-07 | 1.17e-05 |
| *v*-*_S_*_3_ | *k_F_*_4_ = 0.1 min^-1^ | 1.55e-07 | 3.34e-06 |
| *v_S_*_4_ | *k_S_*_4_ = 0.07 min^-1^ | 5.44e-07 | 1.169e-05 |
| *vS_dket* | *kS_dket* = 0.01 min^-1^ | 2.35e-13 | 5.06e-12 |

**Part III: Figures**

**S1. Reactions Included in Liver Component of the Model ([1])**

**Figure S1. Reactions Included in Liver Component of the Model.** The corresponding rate of each reaction is described in the text.

**S2. Reactions Included in Liver Component of the Model ([1])**

**Figure S2. Reactions Included in the Fat Component of the Model.** The corresponding rate of each reaction is described in the text.

**S3. Reactions Included in Muscle Component of the Model ([1])**

**Figure S3. Reactions Included in the Muscle Component of the Model.** The corresponding rate of each reaction is described in the text.

**S4. Reactions Included in Muscle Component of the Model ([1])**

**Figure S4. Reactions for Transport Across the Cell Membranes.** The corresponding rate of each reaction is described in the text.

**S5. Glucose feeding function**


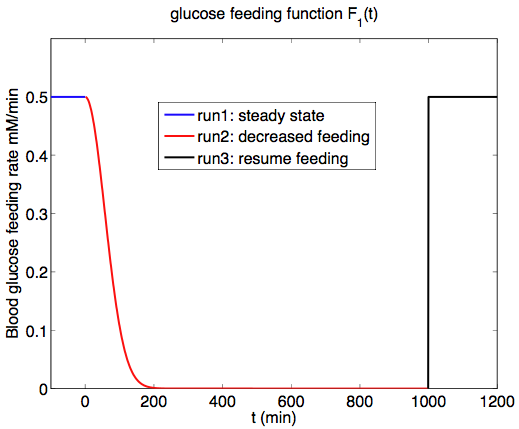


**Figure S5. Glucose feeding function in the blood.** The first run is for the system to reach fed steady state with a constant feeding rate in the blood; the second run is the post-adsorption state with a decreasing glucose feeding rate from the fed steady state (drops below 5% in 140 mins.) The third run is for a glucose stimulus to enter the system as a step function.

**S6. Glycogen concentration in fed and fasted livers.**


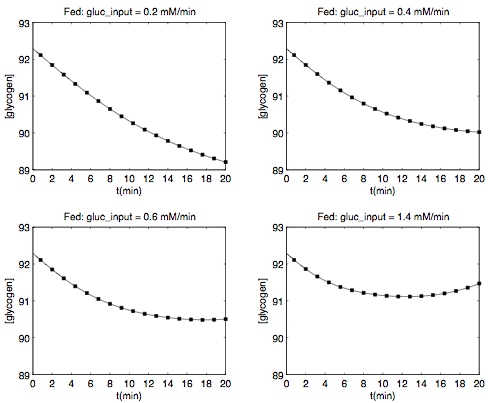

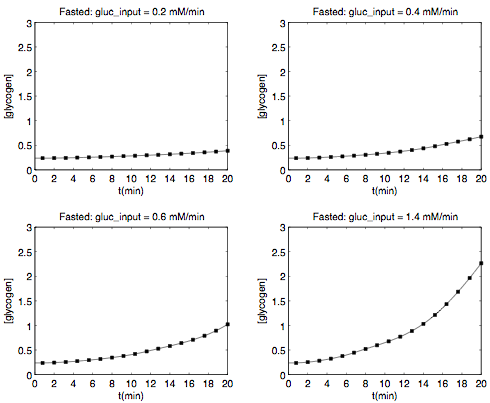


(a) Glycogen concentration in fed livers after a glucose stimulus enters blood stream at t=0.

(b) Glycogen concentration in fasted livers after a glucose stimulus enters blood stream at t=0.

**Figure S6. Glycogen concentrations in fed and fasted livers.**

**Part IV: Major Enzymes/Signaling Proteins**

**Table S11. The enzyme/protein names, abbreviations and the reactions are based on KEGG pathway.**

| **ENZYME** (Enzyme Commission number and name)  REACTION |
| --- |
| **HK** (EC 2.7.1.1 hexokinase)  **GLK** (EC 2.7.1.2 glucokinase)  ATP + glucose = ADP + glucose 6-phosphate |
| **PK** (EC 2.7.1.40 pyruvate kinase)  ADP + phosphoenolpyruvate = ATP + pyruvate |
| **LDH** (EC 1.1.1.27 lactate dehydrogenase)  pyruvate + NADH + H+ = lactate + NAD+ |
| **PDH** (EC 1.2.1.51 pyruvate dehydrogenase)  pyruvate + CoA + NAD+ = acetyl-CoA + CO2 + NADH |
| **PC**  (EC 6.4.1.1 pyruvate carboxylase)  ATP + oxaloacetate = ADP + phosphoenolpyruvate + CO2 |
| **CS** (EC 2.3.3.8 citrate synthase)  ADP + phosphate + acetyl-CoA + oxaloacetate = ATP + citrate + CoA |
| **MDH** (EC 1.1.1.37 malate dehydrogenase)  malate + NAD+ = oxaloacetate + NADH + H+ |
| **MS** (malate shuttle)  transports malate across mitochondrial membrane |
| **ALT** (EC 2.6.1.2 alanine transaminase)  alanine + α-ketoglutarate = pyruvate + glutamate |
| **AGT** (EC 2.6.1.44 L-alanine-glycine transaminase)  L-alanine + glyoxylate = pyruvate + glycine |
| **PEPCK** (EC 4.1.1.49 PEP carboxykinase)  ATP + oxaloacetate = ADP + phosphoenolpyruvate + CO2 |
| **G6Pase** (EC 3.1.3.9 glucose-6-phosphatase)  glucose 6-phosphate + H2O = glucose + phosphate |
| **GS** (EC 2.4.1.11 glycogen synthase)  UDP-glucose + (1,4-glucosyl)n = UDP + (1,4-glucosyl)(n+1) |
| **GP** (EC 2.4.1.1 glycogen phosphorylase)  ATP + glucose = ADP + glucose 6-phosphate |
| **CL** (EC 2.3.3.8 ATP citrate lyase)  ATP + citrate + CoA = ADP + phosphate + acetyl-CoA + oxaloacetate |
| **AC**  (EC 6.4.1.2 acetyl-CoA carboxylase)  ATP + acetyl-CoA + HCO3- = ADP + phosphate + malonyl-CoA |
| **FASN** (EC 2.3.1.85 Fatty Acid Synthase)  acetyl-CoA + n malonyl-CoA + 2n NADPH + 2n H+ = a long-chain fatty acid + (n+1) CoA + n CO2 + 2n NADP+ |
| **ACS** (EC 6.2.1.3 acyl-CoA synthetase)  ATP + a long-chain carboxylic acid + CoA = AMP + diphosphate + an acyl-CoA |
| **CPT1** (EC:[2.3.1.21](http://www.genome.jp/dbget-bin/www_bget?ec:2.3.1.21) carnitine O-palmitoyltransferase 1)  palmitoyl-CoA + L-carnitine = CoA + L-palmitoylcarnitine |
| **HMGS** (EC 2.3.3.10 HMG-CoA synthase)  acetyl-CoA + H2O + acetoacetyl-CoA = 3-hydroxy-3-methylglutaryl-CoA + CoA |
| **DGAT** (EC 2.3.1.20 diacylglycerol O-acyltransferase)  acyl-CoA + 1,2-diacyl-sn-glycerol = CoA + triacylglycerol |
| **LPL** (EC 3.1.1.34  lipoprotein lipase)  triacylglycerol + H2O = diacylglycerol + a carboxylate |
| **PP-1** (EC 3.1.3.16 protein phosphatase-1)  a phosphoprotein + H2O = a protein + phosphate |
| **GSP** (EC 3.1.3.42 [glycogen-synthase-D] phosphatase)  [glycogen-synthase D] + H2O = [glycogen-synthase I] + phosphate |
| **CAPK** cAMP dependent protein kinase |
| **PK** (phosphorylase kinase) |
| **INS** (Insulin) |
| **GCG** (Glucagon) |
| **cAMP** (Cyclic adenosine monophosphate) |

References:

1. Todd, A.J., *Inclusion of a glycogen regulation mathematical model into a contextual metabolic framework.* Ph.D. Thesis, 2008, University of North Carolina at Chapel Hill.

2. Tu, J. and B.E. Tuch, *Glucose regulates the maximal velocities of glucokinase and glucose utilization in the immature fetal rat pancreatic islet.* Diabetes, 1996. **45**(8): p. 1068-75.

3. Jitrapakdee, S., M.E. Walker, and J.C. Wallace, *Functional expression, purification, and characterization of recombinant human pyruvate carboxylase.* Biochem Biophys Res Commun, 1999. **266**(2): p. 512-7.

4. Guionie, O., et al., *Identification and characterisation of a new human glucose-6-phosphatase isoform.* FEBS Lett, 2003. **551**(1-3): p. 159-64.

5. Jeng, J., et al., *Pyruvate dehydrogenase E1 alpha isoform in rat testis: cDNA cloning, characterization, and biochemical comparison of the recombinant testis and liver enzymes.* Comp Biochem Physiol B Biochem Mol Biol, 1998. **120**(1): p. 205-16.

6. Huang, K.P. and J.C. Robinson, *Purification and properties of the glucose-6-phosphate-dependent form of human placental glycogen synthase.* Arch Biochem Biophys, 1976. **175**(2): p. 583-9.

7. Medicus, R. and J. Mendicino, *Role of enzyme interactions in the regulation of glycolysis and gluconeogenesis. Purification and properties of the phospho- and dephospho-forms of glycogen phosphorylase from swine kidney.* Eur J Biochem, 1973. **40**(1): p. 63-75.

8. Nicolau, J., D.N. Souza, and G. Nunez-Burgos, *Regulation of phosphofructokinase-1 on submandibular salivary glands of rats after isoproterenol administration.* Arch Physiol Biochem, 2000. **108**(5): p. 437-43.

9. Adams, A., C. Redden, and S. Menahem, *Characterization of human fructose-1,6-bisphosphatase in control and deficient tissues.* J Inherit Metab Dis, 1990. **13**(6): p. 829-48.

10. Srivastava, L.K. and N.Z. Baquer, *Purification and properties of rat brain pyruvate kinase.* Arch Biochem Biophys, 1985. **236**(2): p. 703-13.

11. LeVan, K.M. and E. Goldberg, *Properties of human testis-specific lactate dehydrogenase expressed from Escherichia coli.* Biochem J, 1991. **273 ( Pt 3)**: p. 587-92.

12. Mendiola, P. and J. De Costa, *The effects of temperature and pH on the kinetic properties of heart muscle lactate dehydrogenase from anuran amphibians.* Comp Biochem Physiol B Biochem Mol Biol, 1991. **98**: p. 529-534.

13. Ruscak, M., J. Orlicky, and V. Zubor, *Isoelectric focusing of the alanine aminotransferase isoenzymes from the brain, liver and kidney.* Comp Biochem Physiol B, 1982. **71**(1): p. 141-4.

14. DeRosa, G. and R.W. Swick, *Metabolic implications of the distribution of the alanine aminotransferase isoenzymes.* J Biol Chem, 1975. **250**(20): p. 7961-7.

15. Ward, C.W. and P.J. Schofield, *Glycolysis in Haemonchus contortus larvae and rat liver.* Comp Biochem Physiol, 1967. **22**(1): p. 33-52.

16. Gubern, G., et al., *Subcellular distribution of alanine aminotransferase activity in human liver.* Biochem Soc Trans, 1990. **18**(6): p. 1287-8.

17. Mutalik, V.K. and K.V. Venkatesh, *Quantification of the glycogen cascade system: the ultrasensitive responses of liver glycogen synthase and muscle phosphorylase are due to distinctive regulatory designs.* Theor Biol Med Model, 2005. **2**: p. 19.

18. Ingebritsen, T.S. and P. Cohen, *Protein phosphatases: properties and role in cellular regulation.* Science, 1983. **221**(4608): p. 331-8.

19. Foulkes, J.G., et al., *A kinetic analysis of the effects of inhibitor-1 and inhibitor-2 on the activity of protein phosphatase-1.* Eur J Biochem, 1983. **132**(2): p. 309-13.

20. Nimmo, G.A. and P. Cohen, *The regulation of glycogen metabolism. Phosphorylation of inhibitor-1 from rabbit skeletal muscle, and its interaction with protein phosphatases-III and -II.* Eur J Biochem, 1978. **87**(2): p. 353-65.

21. Shacter, E., P.B. Chock, and E.R. Stadtman, *Energy consumption in a cyclic phosphorylation/dephosphorylation cascade.* J Biol Chem, 1984. **259**(19): p. 12260-4.

22. Meinke, M.H. and R.D. Edstrom, *Muscle glycogenolysis. Regulation of the cyclic interconversion of phosphorylase a and phosphorylase b.* J Biol Chem, 1991. **266**(4): p. 2259-66.

23. Killilea, S.D., et al., *Evidence for the coordinate control of activity of liver glycogen synthase and phosphorylase by a single protein phosphatase.* J Biol Chem, 1976. **251**(8): p. 2363-8.

24. Cohen, P., *Control of enzyme activity*. 2nd ed. 1983, London; New York: Chapman and Hall. 96 p.

25. Aiston, S., et al., *Glucose 6-phosphate causes translocation of phosphorylase in hepatocytes and inactivates the enzyme synergistically with glucose.* Biochem J, 2004. **377**(Pt 1): p. 195-204.

26. Beavo, J.A., P.J. Bechtel, and E.G. Krebs, *Activation of protein kinase by physiological concentrations of cyclic AMP.* Proc Natl Acad Sci U S A, 1974. **71**(9): p. 3580-3.
