# Supplementary material for: A Whole-Body Model for Glycogen Regulation Reveals a Critical Role for Substrate Cycling in Maintaining Blood Glucose Homeostasis
Source: PLoS Comput Biol. 2011 Dec 1;7(12):e1002272. doi: 10.1371/journal.pcbi.1002272 (PMC3233304; doi:10.1371/journal.pcbi.1002272)
Supplement: Protocol S3 — Readme file for the MATLAB package. (DOCX) [file pcbi.1002272.s003.docx]

**README**

**Required Software to Run Liver_glycogen_model:**

**MATLAB WinRAR(Windows) UnRarX (Mac OS X)**

**How to Run Liver_glycogen_model:**

1. Download and unrar Liver_glycogen_model.rar.
2. Open runmain.m under MATLAB editor and click on ‘run’ button; or type ‘runmain’ in the command window.
3. In runmain.m, two functions are called: main.m is the main program file and plot_conc.m is the plot function for generating enzyme (glycogen synthase a and glycogen phosphorylase a) and substrate (glycogen) concentrations over time. The four concentrations 0.2 mM/min, 0.4 mM/min, 0.6 mM/min and 1.4 mM/min are consistent with those presented in our paper.
4. main.m takes two inputs: ‘state’ and ‘gluc_input’. ‘state’ takes the value of ‘fed’ or ‘fasted’, which determines the fasting time from fed steady state. There are 3 runs in main.m: the first run is for the system to reach fed steady state with a constant feeding rate in the blood; the second run is the post-adsorption state with a decreasing glucose feeding rate from the fed steady state (drops below 5% in 140 mins.) Here, two fasting times are chosen to represent a fed and fasted livers: tmax = 250 mins or tmax = 1200 mins. The third run is for a glucose stimulus to enter the system as a step function. This rate is stored in the input variable ‘gluc_input’.
5. plot_conc.m generates figures for GPa, GSa and glycogen after the glucose stimulus enters the system.
6. Data are stored in the folder ‘./results’ in the form of ‘y_fasted_XpX.mat’ or ‘y_fed_XpX.mat’ where ‘XpX’ is the glucose feeding rate in the blood. For example, ‘y_fasted_0p2.mat’ means glucose input rate = 0.2 in a fasted liver.

UNC COPYRIGHT AND PERMISSION NOTICE

# UNC Software: << *Liver_glycogen_model* >>

Copyright (C) 2009 The University of North Carolina at Chapel Hill
All rights reserved.

The University of North Carolina at Chapel Hill (“UNC”) and the developers (“Developers”) of << *Liver_glycogen_model* >> (“Software”) give recipient (“Recipient”) and Recipient’s Institution (“Institution”) permission to use and copy the software in source and binary forms, with or without modification for **non-commercial purposes only** provided that the following conditions are met:

1. All copies of Software in binary form and/or source code, related documentation and/or other materials provided with the Software must reproduce and retain the above copyright notice, this list of conditions and the following disclaimer.
2. Recipient and Institution shall not distribute Software to any third parties.
3. The Software is provided “As Is.” The Developers can not guarantee the provision of technical support or consultation for the Software. The Developers may provide a location on a UNC Web Site for Recipients to post comments, questions, and suggestions at some time in the future. Recipient may provide the Developers with feedback on the use of the Software in their research at that time. The Developers and UNC are permitted to use any information Recipient provides in making changes to the Software.
4. Recipient acknowledges that the Developers, UNC and its licensees may develop modifications to Software that may be substantially similar to Recipient’s modifications of Software, and that the Developers, UNC and its licensees shall not be constrained in any way by Recipient in UNC’s or its licensees’ use or management of such modifications. Recipient acknowledges the right of the Developers and UNC to prepare and publish modifications to Software that may be substantially similar or functionally equivalent to your modifications and improvements, and if Recipient or Institution obtains patent protection for any modification or improvement to Software, Recipient and Institution agree not to allege or enjoin infringement of their patent by the Developers, UNC or any of UNC’s licensees obtaining modifications or improvements to Software from the UNC or the Developers.
5. Recipient and Developer will acknowledge in their respective publications the contributions made to each other’s research involving or based on the Software. The current citations for Software are:

*<<Pending.>>>*

1. Any party desiring a license to use the Software for commercial purposes shall contact The Office of Technology Development at UNC at 919-966-3929.

THIS SOFTWARE IS PROVIDED BY THE COPYRIGHT HOLDERS, CONTRIBUTORS, AND THE UNIVERSITY OF NORTH CAROLINA AT CHAPEL HILL "AS IS" AND ANY EXPRESS OR IMPLIED WARRANTIES, INCLUDING, BUT NOT LIMITED TO, THE IMPLIED WARRANTIES OF MERCHANTABILITY AND FITNESS FOR A PARTICULAR PURPOSE ARE DISCLAIMED. IN NO EVENT SHALL THE COPYRIGHT OWNER, CONTRIBUTORS OR THE UNIVERSITY OF NORTH CAROLINA AT CHAPEL HILL BE LIABLE FOR ANY DIRECT, INDIRECT, INCIDENTAL, SPECIAL, EXEMPLARY, OR CONSEQUENTIAL DAMAGES (INCLUDING, BUT NOT LIMITED TO, PROCUREMENT OF SUBSTITUTE GOODS OR SERVICES; LOSS OF USE, DATA, OR PROFITS; OR BUSINESS INTERRUPTION) HOWEVER CAUSED AND ON ANY THEORY OF LIABILITY, WHETHER IN CONTRACT, STRICT LIABILITY, OR TORT (INCLUDING NEGLIGENCE OR OTHERWISE) ARISING IN ANY WAY OUT OF THE USE OF THIS SOFTWARE, EVEN IF ADVISED OF THE POSSIBILITY OF SUCH DAMAGE.
